# An evolutionary conserved interaction between the Gcm transcription factor and the SF1 nuclear receptor in the female reproductive system

**DOI:** 10.1038/srep37792

**Published:** 2016-11-25

**Authors:** Pierre B. Cattenoz, Claude Delaporte, Wael Bazzi, Angela Giangrande

**Affiliations:** 1 Institut de Génétique et de Biologie Moléculaire et Cellulaire, ILLKIRCH, France; 2 Centre National de la Recherche Scientifique, UMR7104, ILLKIRCH, France; 3 Institut National de la Santé et de la Recherche Médicale, U964, ILLKIRCH, France; 4 Université de Strasbourg, ILLKIRCH, France

## Abstract

NR5A1 is essential for the development and for the function of steroid producing glands of the reproductive system. Moreover, its misregulation is associated with endometriosis, which is the first cause of infertility in women. Hr39, the *Drosophila* ortholog of NR5A1, is expressed and required in the secretory cells of the spermatheca, the female exocrine gland that ensures fertility by secreting substances that attract and capacitate the spermatozoids. We here identify a direct regulator of Hr39 in the spermatheca: the Gcm transcription factor. Furthermore, lack of Gcm prevents the production of the secretory cells and leads to female sterility in *Drosophila.* Hr39 regulation by Gcm seems conserved in mammals and involves the modification of the DNA methylation profile of *mNr5a1*. This study identifies a new molecular pathway in female reproductive system development and suggests a role for hGCM in the progression of reproductive tract diseases in humans.

In mammals and insects, the process of spermatozoid maturation occurs first in the male before copulation and second after copulation where molecules secreted by the female reproductive tract epithelium preserve and capacitate the spermatozoids^1^,^2^,^3^,^4^. Capacitation is primordial for fertilisation and spermatozoids are viable in the female reproductive tract for several days in human^5^, several years in honey bees^6^,^7^ and several decades in ants^8^. In both mammals and insects, the inability to capacitate/store the spermatozoids has a strong impact on female fertility^9^,^10^,^11^,^12^,^13^,^14^,^15^.

Several insect species have developed specific structures called spermathecae that preserve the spermatozoids well after copulation in the female reproductive tract. The molecules that attract, store and capacitate the spermatozoids in the spermatheca are produced by a layer of secretory cells (SC)^12^. The hormone receptor Hr39 allows the generation of the SC, hence ensuring female fertility. Two mammalian orthologs of Hr39 have been described. The nuclear receptor 5A2 (NR5A2 also known as LRH-1) was associated with pre-eclampsia in humans and is involved in cell proliferation, bile acid metabolism and steroidogenesis^16^,^17^. The nuclear receptor 5A1 (NR5A1 also known as SF-1) (human *NR5A1* gene is *hNR5A1* and mouse ortholog is *mNr5a1* throughout the text)^14^,^15^ is involved in the development and in the function of the pituitary gland, of the adrenal gland and of the gonads^18^,^19^. Its mutation leads to severe defects in sexual organ formation and its misexpression is associated with changes in its DNA methylation profile and with endometriosis, the major cause of infertility in women^20^,^21^,^22^,^23^.

In this study, we identify the zinc finger transcription factor Glial cells missing (Gcm also known as Glial cell deficient or Glide) as a major transcriptional regulator of *Hr39* during *Drosophila* spermatheca development. While the complete lack of Gcm leads to embryonic lethality due to the loss of glia^24^, we show that its partial lack is compatible with life and leads to almost complete sterility in females. In addition, clones of cells completely lacking Gcm in the spermatheca are devoid of SC. We show that Gcm controls the differentiation of the SC by controlling the expression of Hr39 directly. Such transcriptional control seems evolutionarily conserved, as the Gcm murine orthologs (mGCM1 and mGCM2), which were described for their expression in placenta, parathyroid, thymus, kidney and nervous system^25^,^26^,^27^,^28^,^29^, are also expressed in the uterus. Finally, assays in cells indicate that the Gcm family promotes the expression of mNR5A1/hNR5A1 and that the mGCM proteins induce the same changes in the DNA methylation of the *hNR5a1* locus as those observed in endometriosis.

Collectively, our data reveal the regulatory pathway underlying SC differentiation in the *Drosophila* spermatheca and the conserved regulation of Hr39 and NR5A1, which represents the first evidence of the functional conservation of the Gcm transcription factors. Understanding the regulation of Hr39 expression may shed light on the physiopathological mechanisms of the major cause of infertility in women.

## Results

### Gcm is required for female fertility and is expressed in the *Drosophila* spermatheca

**Figure 1:**
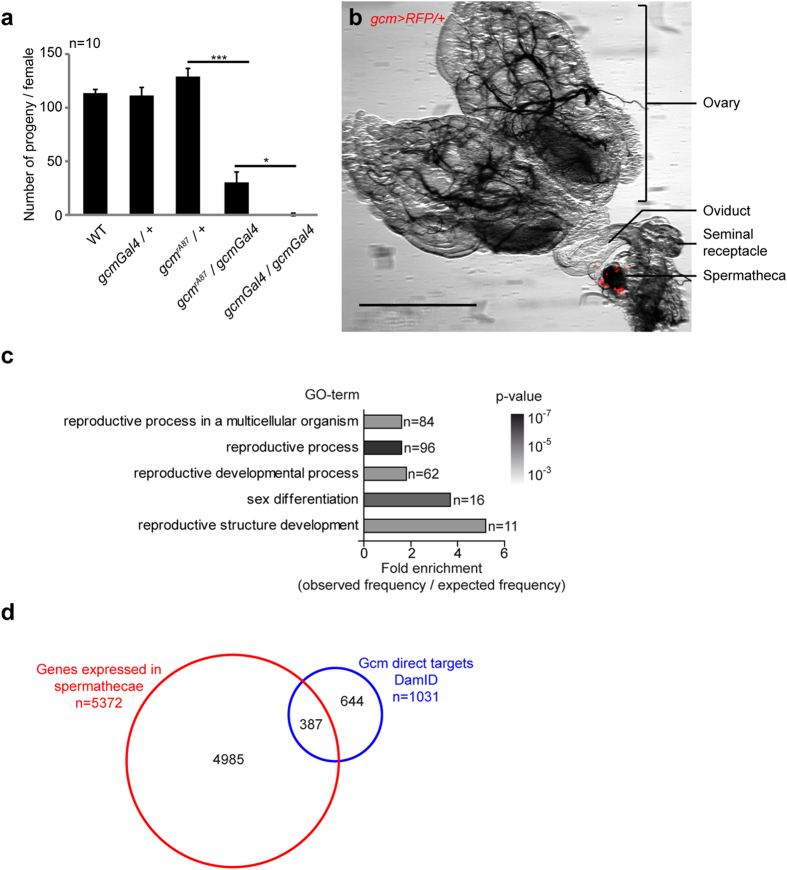
Gcm is expressed in the spermatheca and controls fertility. (**a**) Fertility assays carried out on *gcm* hypomorphs. The histogram shows the average number of progenies per female of the following genotypes: wild type (WT), *gcmGal4/*+ and *gcm*^*rA87*^/+, which represent the control strains, as well as *gcmGal4/gcm*^*rA87*^ and *gcmGal4/gcmGal4*, which represent *gcm* hypomorphic conditions. Ten crosses were made per genotype (n = 10). The error bars represent standard errors of the mean (s.e.m.). Student test was used to calculate the p-values: >0.05 = ns; <0.05–0.01 <= *; <0.01–0.001 <= **; <0.001 = ***. (**b**) Reproductive system of an adult control female (*gcmGal4/*+*;UAS-RFP*). Overlay of the images taken with white light and by epifluorescence (561nm). The scale bar represents 500 μm. (**c**) GO-term enrichment analysis of the genes directly targeted by Gcm according to a DamID screen^39^. The histogram represents the fold enrichments obtained for GO-terms linked to reproduction (enrichment >1.5, FDR <2%, p-value < 10^−3^), n = number of genes. (**d**) Overlap between the direct targets of Gcm according to a DamID screen (blue) and the genes expressed in spermatheca according to a spermatheca transcriptome^13^.

In *Drosophila*, the complete lack of the Gcm protein leads to embryonic lethality due to the transformation of glial cells into neurons^24^,^30^,^31^. Viable hypomorphic mutations, however, allow the analysis of *gcm* mutant animals at later stage^24^,^31^,^32^,^33^: the *gcm*^*rA87*^ allele is due to the insertion of a P-element containing the *LacZ* gene in the promoter of *gcm* and the *gcmGal4* allele has been produced upon replacement of the *LacZ* by the *Gal4* gene^24^,^31^,^32^,^33^. The *gcmGal4* homozygous and the transheterozygous *gcmGal4/gcm*^*rA87*^ animals reach adulthood and display fertility defects. To assess whether the defects are sex specific, we crossed wild type (WT, *Oregon-R)* males with *gcmGal4* homozygous or with transheterozygous females and found a significantly reduced number of offspring compared to that obtained in control crosses (<1% and 20% of the progeny, respectively, Fig. 1a). In contrast, fertility assays on transheterozygous males showed no fertility defects (data not shown). Thus, Gcm is required in reproduction in females, in addition to its well-known role in glia and blood development^24^,^30^,^31^,^34^,^35^,^36^,^37^,^38^.

To clarify the role of Gcm on fertility, we crossed the *gcmGal4* driver, which faithfully mimics the expression of Gcm^32^,^33^, with a *UAS-RFP* reporter. RFP expression was detected in the adult spermatheca, while no labelling was observed in the ovaries nor in the oviduct (Fig. 1b). Gcm expression in the adult spermatheca was confirmed by qPCR assays (Supplemental Figure S1).

Finally, a GO-term analysis on a genome-wide screen aiming at identifying the direct targets of Gcm^39^ specifically highlighted the genes involved in the reproductive system development as the most enriched class of genes after those involved in nervous system development, in line with the known role of Gcm at the glial determinant^24^,^30^,^31^ (Fig. 1c). Comparison between this screen and the published transcriptome of the spermatheca^13^ revealed that 387 direct targets of Gcm are expressed in this organ (Fig. 1d, list in Supplemental Table S1).

This data indicate that Gcm is necessary for female fertility and that it is expressed in the spermatheca.

### The *gcm* mutation affects the secretory cells of the spermathecae

**Figure 2:**
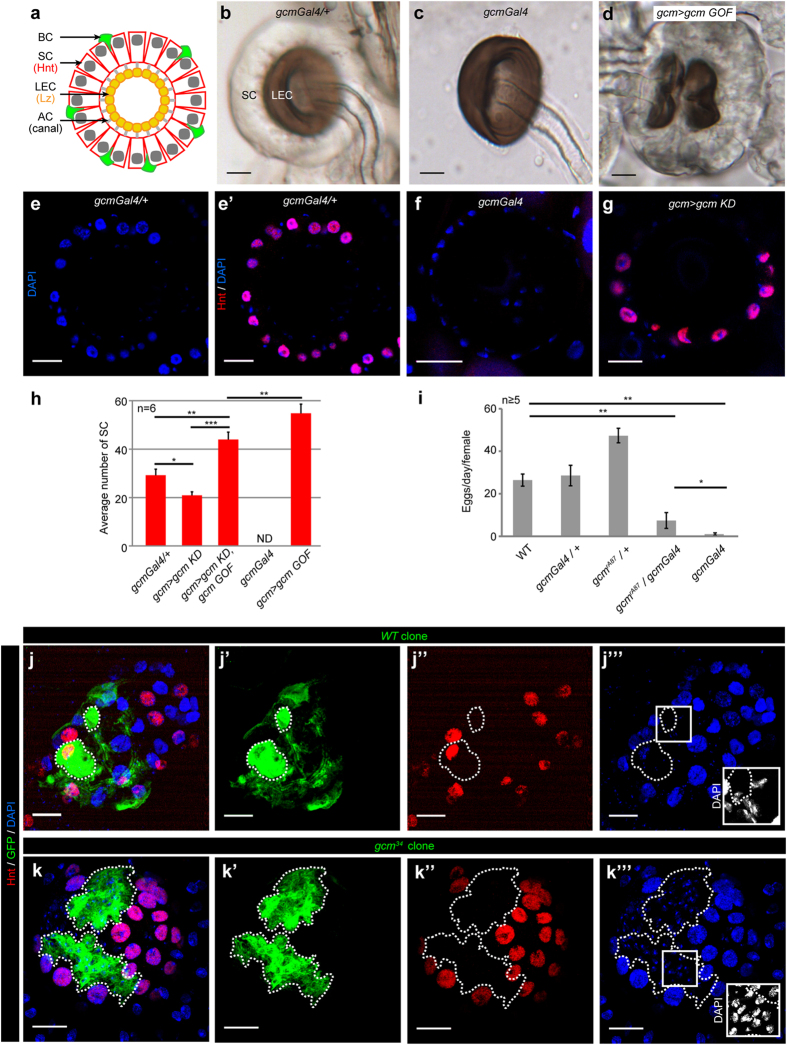
Gcm is involved in the development of the secretory cells of the spermatheca. (**a**) Schematic representation of an adult spermatheca cross-section. The SC express Hindsight (Hnt) and the LEC Lozenge (Lz). (**b–d**) Spermathecae analysed by bright-field microscopy. The spermathecae were dissected from adult females (1 to 3-day-old) *gcmGal4/*+ (control) (**b**), *gcmGal4* (**c**), and *gcmGal4/*+*;UAS-gcm/*+ (*gcm* > *gcm GOF*) **(d)**. Unless otherwise specified, all scale bars here and in the following figures represent 20 μm. (**e–g**) Single optical sections of spermathecae analysed by confocal microscopy from adult females of the following genotypes: *gcmGal4/*+ (**e,e’**), *gcmGal4* (**f**) and *gcmGal4/*+*;UAS-gcmRNAi/*+ (*gcm* > *gcm KD*) (**g**) labelled with anti-Hnt (Hnt, in red) and DAPI (blue). (**e**) and (**e’**) represent the DAPI and the overlap of DAPI and anti-Hnt labelling of the *gcmGal4/*+ spermatheca, respectively. (**h**) Average number of secretory cells counted in cross-sections of adult spermathecae of the indicated genotypes (see materials and methods). At least 6 spermathecae were analysed per genotype, the error bars and p-values are as described for Fig. 1a. (**i**) Number of eggs laid per female and per day for the indicated genotypes. At least five replicates were made per genotype. (**j–k”’**) MARCM clonal analysis in a *gcm* mutant background. The images represent full projections of spermathecae analysed by confocal microscopy from adult females showing WT (**j–j”’**) or *gcm*^*34*^ mutant (**k–k”’**) clones. The spermathecae were labelled with anti-GFP (the clones express GFP, in green), anti-Hnt (Hnt, in red) and DAPI (blue) (**j,k**), the clones are indicated by dashed lines. Each marker is shown individually in (**j’** and **k’**) for anti-GFP, (**j”** and **k”**) for anti-Hnt and (**j”’** and **k”’**) for DAPI. The insets in (**j”’** and **k”’**) show a higher magnification of the nuclei with the DAPI in grey. See also Supplemental Figures S2 and S4.

Two elegant studies^14^,^40^ showed that the spermatheca of *Drosophila* contains a layer of lumen epithelial cells (LEC) expressing the Runt-domain transcription factor Lozenge (Lz), which is essential for the development of the whole spermatheca^14^ (Fig. 2a). Surrounding the LEC is the layer of SC that express and require the transcription factor Hindsight (Hnt)^15^. Accessory cells are located basal (basal cells, BC) to the SC and apical (apical cells, AC) to the LEC. The AC are thought to secrete a cuticular canal that connects the secretory unit to the lumen of the spermatheca, which contains the spermatozoids. AC and BC undergo apoptosis during pupal spermatheca development, with some BC being still present in young adult females^14^.

To assess the mode of action of Gcm, we analysed the morphology of the spermathecae in animals carrying altered levels of Gcm. The WT SC appear as a translucent layer of cells surrounding a dark cuticular structure that is produced by the LEC (Fig. 2b). In hypomorphic *gcm* conditions (*gcmGal4* homozygous animals), the SC layer is completely absent, leaving the dark cuticular structure relatively unaffected (Fig. 2c). Accordingly, immunolabelling assays show a complete lack of SC in homozygous *gcmGal4* females (Fig. 2f,h), which leads to the absence of spermatozoids in the spermathecae (Supplemental Figures S2a–d). The lack of SC in *gcm* homozygous females is also observed in other hypomorphic *gcm* conditions such as transheterozygous *gcmGal4/gcm*^*rA87*^ animals and can be rescued by overexpressing Gcm (Supplemental Figures S2e–g). Of note, some spermathecae from transheterozygous *gcmGal4/gcm*^*rA87*^ animals show few remaining SC (Supplemental Figure S2h’), explaining why this strain is not completely sterile. In addition, the number of SC significantly decreases when Gcm is knocked-down by RNAi (*gcm KD*) using the *gcmGal4* as a driver (Fig. 2g,h). The egg laying rate is in agreement with this data. A positive correlation was previously made between the number of SC of the spermathecae and the number of eggs laid^15^ and indeed the number of SC as well as the egg laying rate decrease in *gcm* hypomorphs (Fig. 2h,i). The reduction in SC number no longer persists in *gcm KD* spermathecae that also carry the *UAS-gcm* transgene. Indeed, these spermathecae carry supernumerary SC (Fig. 2h), suggesting that Gcm expression may be sufficient to induce the differentiation of the SC.

Finally, to analyse the phenotype of a null *gcm* allele, MARCM clones were produced using the *Df*(*2L*)*132* strain in which the *gcm* gene is completely deleted^33^,^41^ (Supplemental Figure S2i). Similar clonal analyses were also performed using a *gcm* hypomorphic but lethal mutation induced by P-element mutagenesis, *gcm*^*34 *^24 (Fig. 2j–k”’). Recombination was induced at the 3^rd^ instar larval stage prior to spermatheca differentiation. WT clones contain both cell types (SC and LEC), whereas *Df(2L)132* and *gcm*^*34*^ mutant clones contain LEC but completely lack SC. Thus, Gcm is necessary for the differentiation of SC. Given the strong phenotype observed in loss of function *gcm* alleles, we assessed the consequences of overexpressing Gcm in its own territory of expression in WT animals (*gcm* > *gcm GOF*). In these gain of function (GOF) animals, the dark cuticular structure and the LEC are present but the morphology of the spermatheca is altered (Fig. 2d, Supplemental Figures S2j-j”). In addition, these spermathecae display a very high number of SC (Fig. 2h).

Altogether, this data clearly indicate that Gcm is expressed and required in the spermatheca to control SC differentiation.

### Gcm is expressed in the precursors of the secretory cells

**Figure 3:**
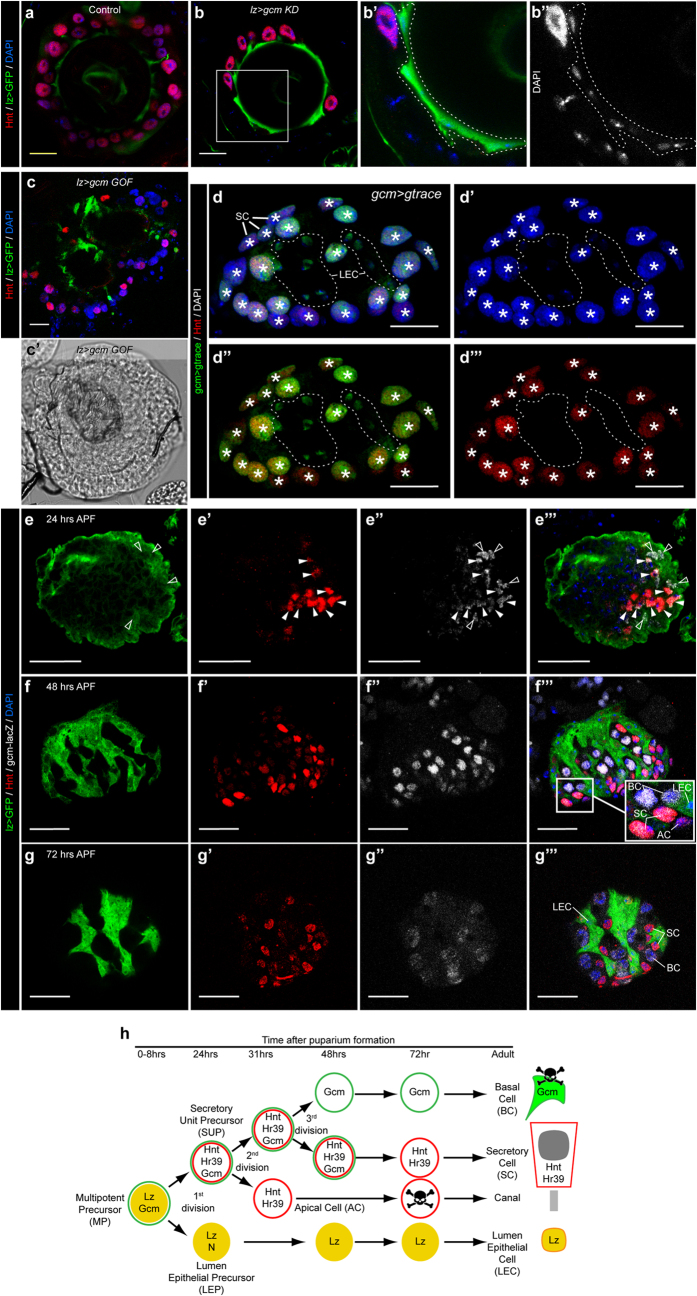
Gcm is expressed early in the secretory cell precursor to initiate the differentiation of the secretory cell. (**a–c**) Single confocal sections of spermathecae from adult females (1 to 3-day-old) *lzGal4,UAS-mCD8GFP/*+ (control) (**a**), *lzGal4,UAS-mCD8GFP/*+*;UAS-gcmRNAi* (*lz* > *gcm KD*) (**b**) and *lzGal4,UAS-mCD8GFP/*+*;UAS-gcm* (*lz* > *gcm GOF*) **(c)** labelled with anti-GFP (*lz* > *GFP*, in green), anti-Hnt (Hnt, in red) and DAPI (blue). The region indicated by the white square in (**b**) is magnified in (**b’**) and (**b”**), in which the LEC are indicated by a dashed line. DAPI is in grey in (**b”**). (**c’**) Bright-field image of a *lz* > *gcm GOF* spermatheca. (**d–d”’**) Confocal projection of a *gcmGal4/*+*;g-trace/*+ (*gcm* > *g-trace*) adult spermatheca labelled with anti-Hnt (Hnt, in red), anti-GFP (*gcm* > *g-trace*, in green) and DAPI (blue). (**d**) represents the overlay of anti-Hnt, anti-GFP and DAPI, (**d’**) shows the DAPI labelling, (**d”**) Gcm lineage and Hnt and (**d”’**) anti-Hnt. The white asterisks indicate the SC and the LEC are indicated by a dashed line. (**e–g”’**) Confocal projections of *lzGal4,UAS-mCD8GFP/*+*;gcm*^*rA87*^/+ pupal spermathecae labelled with anti-βgal (*gcm-lacZ*, in grey), anti-Hnt (Hnt, in red), anti-GFP (*lz* > *GFP*, in green) and DAPI (blue). The images were taken at 28 hrs after puparium formation (APF) (**e–e”’**), 48 hrs APF (**f–f”’**) and 72 hrs APF (**g–g”’**). Each marker is shown individually in (**e**–**g**) for anti-GFP, (**e’,f’,g’**) for anti-Hnt, (**e”,f”,g”**) for anti-βgal and the overlay of the three channels and DAPI is shown in (**e”’,f”’,g”’**). The white arrowheads indicate cells expressing Gcm and Hnt, which correspond to the SUP, the empty arrowheads indicate cells expressing Lz and Gcm, which correspond to the MP (**e–e”’**). The inset **(in f”’**) shows SC expressing Hnt and low levels of Gcm, BC expressing high levels of Gcm, an AC expressing Hnt only and an LEC expressing Lz. (**h**) Schematic representation of spermatheca development (modified from ref. 15). The time scale is indicated above the schematic in hours APF. The yellow circles indicate Lz expression, the red circles Hnt and the green circles Gcm expression. The skull pictograms indicate the cells undergoing apoptosis. See also Figure S3.

The mutant phenotype prompted us to assess the role and the mode of action of Gcm. Given the early and transient expression of Gcm in glial cells^24^,^30^,^31^, we analysed the mutant spermathecae and the profile of Gcm expression during development. Spermathecae develop during the pupal stage and the different cell types arise from multipotent precursors (MP) that express Lz and divide to produce the lumen epithelium precursors (LEP) also expressing Lz as well as the secondary precursors called Secretory Unit Precursors (SUP), which do not express Lz^14^. Each SUP divides and produces the AC and a tertiary precursor, which in turn divides and produces the SC as well as the BC that undergoes apoptosis at the adult stage (Fig. 3h)^14^.

First, we knocked down Gcm expression using the *lzGal4* driver (*lz* > *gcm KD*), which is active in the MP. Like in hypomorphic conditions and in *gcm* > *gcm KD* animals, RNAi-mediated down-regulation of Gcm in the MP leads to the decrease of the number of SC in the adult spermatheca (Fig. 3a,b) and the LEC are not impacted (Fig. 3b–b”). The similar phenotypes obtained with *gcm*> and *lz*>, a driver that is not active in the SUP^14^, suggest that the *gcm* promoter is already active in the MP that generates all cell types of the spermatheca (including SC and LEC). We then proceeded to overexpress Gcm under the control of the *lzGal4* driver (*lz* > *gcm GOF*) and found that this leads to severe spermatheca defects including a deformed and almost absent cuticular structure. This phenotype is stronger than the overexpression of Gcm using the *gcmGal4* driver (*gcm* > *gcm GOF*) in which the cuticular structure can still be observed (compare Fig. 3c’ and Supplemental Figures S2j–j”). This indicates that premature Gcm expression prevents LEC development and suggests that Gcm is expressed below threshold levels in the MP.

Following this, we tracked the lineage of the Gcm expressing cells by crossing the *g-trace* flies^42^ with the *gcmGal4* flies and found that both SC and LEC originate from cells expressing Gcm (white asterisks and dashed line, respectively, in Fig. 3d–d”’). In addition, we tracked Gcm expression during spermatheca development using the *gcm*^*rA87*^ βGal reporter in heterozygous conditions. By 24hrs after puparium formation (APF), after the division of the MP, the SUP co-expresses Gcm and Hnt (full arrowheads in Fig. 3e–e”’)^15^ and some MP can still be seen co-expressing Lz and βGal (empty arrowheads in Fig. 3e–e”’). At 48 hrs and 72 hrs APF, three types of cells can be identified: the LEC expressing exclusively Lz, the cells expressing Hnt and low levels of Gcm, which comprise the SC (Fig. 3f”’), and the BC expressing Gcm and almost no Hnt (Fig. 3f,g). Few apoptotic AC can also be detected, expressing Hnt (Fig. 3f”’). This confirms that Gcm and Lz are transcribed in the MP and that Gcm remains expressed in the SUP and its offspring.

Finally, in the adult spermatheca, cell-specific immunolabelling on animals carrying the *gcmGal4* driver and the *UAS-mCD8GFP* reporter (*gcm* > *GFP*, Supplemental Figures S3a, S3c–c”) and anti-βgal labelling on the enhancer trap line *gcm*^*rA87*^ in heterozygous conditions (Supplemental Figure S3b) indicate that Gcm is expressed exclusively in the adult BC. These are the cells that undergo apoptosis^14^ (Supplemental Figures S3c–c”), as shown by the decreased number of labelled cells in old *gcm* > *GFP* spermathecae compared to young ones (Supplemental Figures S3d–e’). Of note, the number of BC decreases in the *gcm* > *gcm GOF* spermathecae that instead present a very high number of SC (Fig. 2h, Supplemental Figure S3f), suggesting that the BC may convert into SC in *gcm* > *gcm GOF* spermathecae.

Collectively, our data show that Gcm starts to be expressed in the MP, specifies SUP differentiation and triggers the differentiation of the SC.

### Gcm induces the expression of Hr39 and triggers secretory cell differentiation

**Figure 4:**
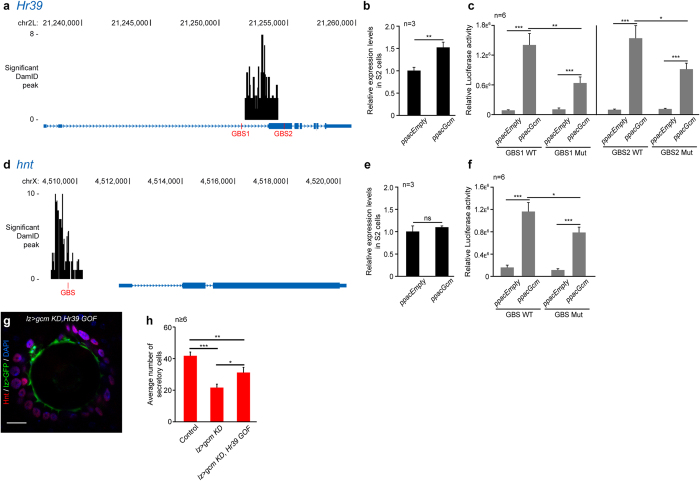
Gcm induces the expression of Hr39 and Hnt. (**a,d**) *Hr39* (**a**) and *hnt* (**d**) loci in the *Drosophila* genome (blue rectangles for exons, blue lines for the introns, the arrowheads indicate the orientation). The canonical Gcm binding sites (GBS) are indicated in red and the black histograms indicate the regions targeted by Gcm^39^. (**b,e**) Expression levels of *Hr39* (**b**) and *hnt* (**e**) measured by qPCR assays in S2 cells transfected with an empty vector (*ppacEmpty*) or with a vector expressing Gcm (*ppacGcm*). The levels are relative to those observed upon transfecting the *ppacEmpty* vector. (**c,f**) Luciferase assays carried out in S2 cells transfected with *ppacEmpty* or with *ppacGcm* and with luciferase vectors carrying the regions covering WT (GBS1 WT and GBS2 WT) or mutated GBS (GBS1 Mut and GBS2 Mut) at the *Hr39* locus **(c)** and the WT or mutated GBS present at the *hnt* locus (**f**). (**g**) Single confocal section of *lzGal4,UAS-mCD8GFP/*+;*UAS-gcmRNAi,UAS-Hr39* (*lz* > *gcm KD,Hr39 GOF*) spermatheca from adult female labelled with anti-GFP (*lz* > *GFP*, green), anti-Hnt (Hnt, in red) and DAPI (blue). (**h**) Average number of SC counted in cross-sections of spermathecae of the indicated genotypes. The *gcm KD* and the *gcm KD,Hr39 GOF* were driven by *lzGal4*. The error bars and p-values (**b**,**c**,**e**,**f and h**) are as described for Fig. 1a. n indicates the number of assays.

Hr39 and Hnt are two transcription factors involved in the development of the spermatheca: knock out as well as *KD* of *hnt* and *Hr39* lead to defective production of SC in the spermatheca^13^,^14^,^15^. In addition, they both contain canonical Gcm binding sites (GBS)^43^,^44^ and were identified as direct targets of Gcm by the genome-wide screen using the DNA adenine methyltransferase identification (DamID) procedure^39^,^45^ (Fig. 4a,d). To validate our data functionally, we analysed the regulation of Hnt and Hr39 by Gcm in S2 cells transfected with a Gcm expression vector. The levels of *Hr39* transcripts are significantly induced by Gcm (Fig. 4b). Next, we built luciferase reporters carrying the two GBS present in the *Hr39* locus where Gcm is binding according to the DamID screen and reporters carrying the mutated GBS. Upon co-transfection with the Gcm expression vector, both GBS present in the *Hr39* locus induce luciferase activity and mutations of either GBS reduces the luciferase expression levels (Fig. 4c), indicating that Gcm induces Hr39 expression through these two GBS (Fig. 4a–c). The endogenous levels of Hnt are not significantly induced by Gcm in S2 cells (Fig. 4e), however, the *hnt* locus possesses one GBS in the promoter region (Fig. 4d) and a luciferase assay similar to that performed on Hr39 indicates a significant induction of *hnt* reporter expression by Gcm, which decreases upon GBS mutagenesis (Fig. 4f). Thus, Gcm is also able to induce the expression of Hnt through the GBS. The lack of induction of the endogenous Hnt in S2 cells is likely due to the absence of cofactors or to the unavailability of the enhancer region targeted by Gcm. In all cases, the mutation of the GBS does not abolish the induction of the luciferase activity completely. This may be due to an indirect effect of Gcm on these promoters or to the presence of non-canonical GBS. Overall, this data indicate that Gcm promotes Hr39 expression and likely contributes to the induction of Hnt expression as well.

Finally, we complemented this data by assessing the biological relevance of the interaction between Gcm and Hr39. Since *gcm KD* in the MP (*lzGal4* driver) leads to a decrease in SC number at adult stage (Fig. 3a,b), we overexpressed Hr39 in *lz* > *gcm KD* spermathecae and found rescue of the mutant phenotype (Fig. 4g,h). The increased number of SC in the adult compared to that observed in animals that only express low levels of Gcm strongly suggests that Hr39 is indeed a major target of Gcm in the development of the female reproductive system. Of note, Hr39 is already detected in the genital discs of the late 3^rd^ instar larvae^13^,^14^ suggesting that the role of Gcm is not to initiate Hr39 expression but to maintain or increase Hr39 expression during the first division of the MP after pupal formation.

### The orthologs of Gcm regulate mNR5A1 expression and are expressed in the mouse uterus

**Figure 5:**
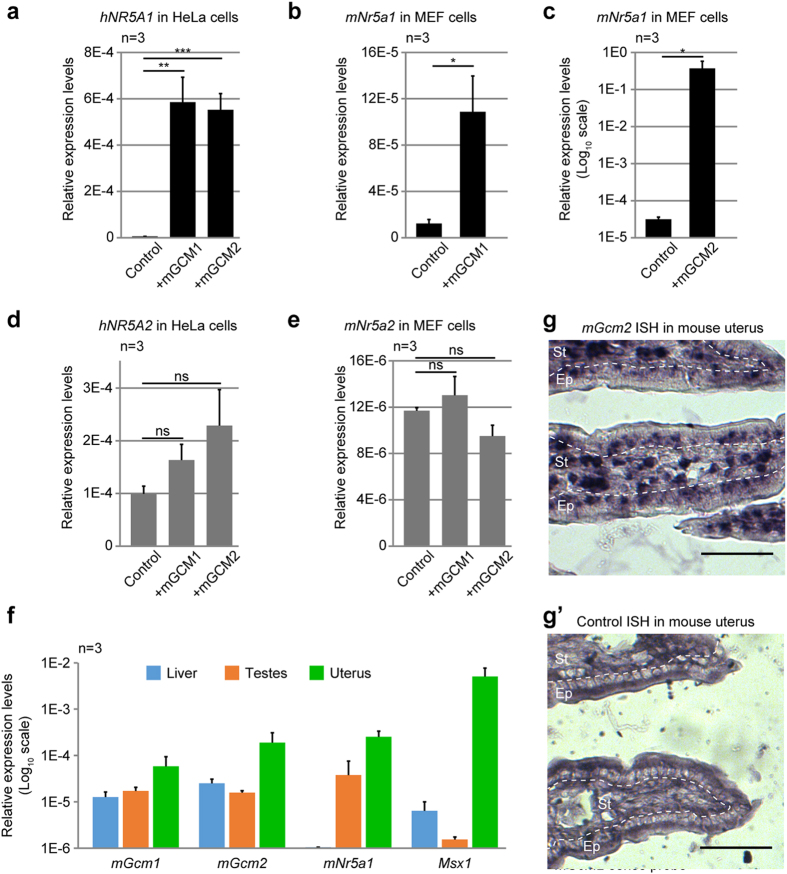
The mGCM proteins induce the expression of the *Hr39* ortholog in mammals. (**a**,**d**) Expression levels of *hNR5A1* (in black (**a**)) and *hNR5A2* (in grey (**d**)) in HeLa cells transfected with an empty vector (Control), an expression vector for mGCM1 (+mGCM1) or an expression vector for mGCM2 (+mGCM2), measured by qPCR. (**b,c,e**) Expression levels of *mNr5a1* (in black, (**b**,**c**) and *mNr5a2* (in grey, (**e**)) in MEF cells transfected with an empty vector (Control) or with an expression vectors for mGCM1 or mGCM2, measured by qPCR. The y-axis is in log_10_ scale in (**c**) and the error bars and p-values are as described for Fig. 1a. n indicates the number of assays. (**f**) Expression levels of *mGcm1*, *mGcm2*, *mNr5a1* and that of the transcription factor *Msx1* in mouse liver, testes and uterus measured by qPCR. The levels are relative to the house-keeping genes *Actb* and *Gapdh*. Each experiment was carried out on three mice. The error bars represent s.e.m. and the y-axis is in log_10_ scale. (**g**,**g’**) *In situ* hybridisation on adult mouse uterus section targeting *mGcm2* using anti-sense *mGcm2* probe (**g**) and negative control using the sense *mGcm2* probe (**g’**). Scale bar represents 50 μm, the stroma (St) of the endometrium corresponds to the area indicated by a dashed line and Ep indicates the columnar epithelium.

The closest mammalian orthologs of the *Hr39* gene are *Nr5a1* and *Nr5a2,* which code respectively for SF-1^13^ and LRH-1^14^ and are both involved in the formation and function of mammalian reproductive tissues^46^,^47^,^48^. We hence assessed whether the functional conservation includes the regulation of *Nr5a1* and *Nr5a2* by the orthologs of Gcm: mGCM1 and mGCM2. First, we measured the endogenous levels of *hNR5A1* and *hNR5A2* in HeLa cells (human) and those of *mNR5A1* and *mNR5A2* in mouse embryonic fibroblasts (MEF) upon transfection of mGCM1 and mGCM2 expression vectors. While the levels of expression of *hNR5A2/mNr5a2* are not modulated by the mGCM proteins, the expression levels of the *hNR5A1*/*mNr5a1* transcripts significantly increase when either mGCM proteins are expressed (Fig. 5a–e). In HeLa cells, both mGCM1 and mGCM2 induce hNR5A1 expression at similar levels (Fig. 5a) and in MEF, mGCM2 induces mNR5A1 expression at higher levels than mGCM1 (9-fold increase compared to WT with mGCM1 versus 6E5-fold increase with mGCM2) (Fig. 5b,c). Then, quantitative PCR (qPCR) analyses indicate that *mGcm1*, *mGcm2* and *mNr5a1* are expressed in the adult mouse uterus and that their levels of expression in this tissue are higher than those found in liver and testes (Fig. 5f). It is important to note, however, that their levels are one order of magnitude lower than the transcription factor *Msx1*, which is known to be strongly active in the uterus^49^ (Fig. 5f). *In situ* hybridisation assays confirm the expression of *mGcm2* mostly in the stroma of the endometrium (Fig. 5g). No signal could be detected using the *mGcm1* probe, likely due to the low levels of *mGcm1* expression it that tissue. This suggests that the regulation of Hr39 expression by Gcm observed in *Drosophila* is conserved in evolution and that mGCM proteins might regulate the expression of mNR5A1 in the mouse uterus.

**Figure 6:**
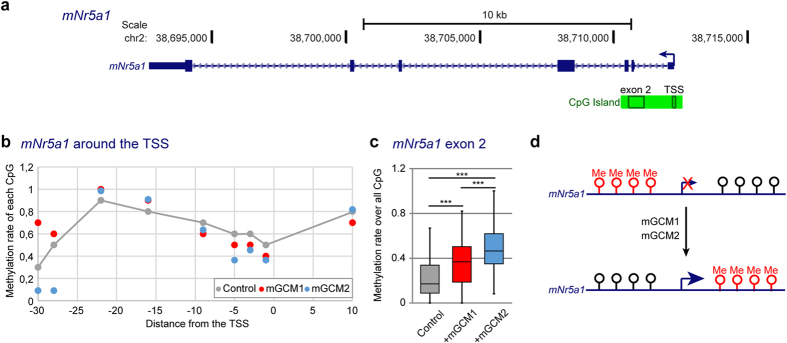
mGCM1 and mGCM2 regulate the methylation profile of *mNr5a1*. (**a**) Schematic representation of the *mNr5a1* locus in the mouse genome. The gene is represented as in Fig. 4a. The genomic coordinates of the locus (genome version mm10) are indicated above the gene. The CpG island is highlighted in green, the rectangles within the CpG island indicate the analysed regions in exon 2 and at TSS. (**b**) Methylation rate for each CpG 30 nucleotides before the TSS and 10 nucleotides after the TSS. The methylation rate in MEF transfected with an empty vector (Control) is indicated in grey, the methylation rate in MEF transfected with an expression vector for mGCM1 is in red and for mGCM2 in blue. Dots above the grey line indicate CpG hypermethylation and dots below indicate CpG hypo-methylation compared to the control cells. (**c**) Box plot representing the distribution of the methylation rate in the CpG island of *mNr5a1* in MEF cells transfected with an empty vector (Control), an expression vector for mGCM1 (+mGCM1) or for mGCM2 (+mGCM2)). The methylation rates were measured for the 51 CpG contained in the exon 2 area highlighted in (**a**) using bisulfite sequencing. The p-values were estimated using paired student test (see materials and methods) and are represented as described in Fig. 1a. (**d**) Schematic representation of the impact of the mGCM protein family on the DNA methylation profile of *mNr5a1*.

Mammalian GCM proteins have been associated with DNA demethylation at the promoter of their target genes: hGCM1 affects *Syncytin 2* demethylation in human placenta^50^ and mGCM1 and mGCM2 affect *Hes5* demethylation in the mouse embryo^29^. For this reason, it was proposed that mGCM proteins trigger DNA demethylation, even though the molecular mode of action was not understood. To further characterize the impact of the *mGcm* genes, we asked whether mNR5A1 regulation by mGCM1 and mGCM2 is associated with changes in the DNA methylation profile of the *mNr5a1* gene using transfected cells. In human and mouse, the *Nr5a1* genes contain a CpG island that covers the transcription start site (TSS) until the 3^rd^ exon (Fig. 6a). The methylation rate of each CpG within the regions covering the 2^nd^ exon and the TSS was estimated by bisulfite sequencing in MEF transfected with an empty vector (Control) or with expression vectors of mGCM1 or mGCM2. The three CpG located around the TSS are demethylated in the presence of mGCM1 or mGCM2 proteins compared to that observed upon transfecting the control plasmid (Fig. 6b). In addition, a significant increase in CpG methylation is observed in the exon 2 region upon mGCM1 or mGCM2 transfection (Fig. 6c). The highest levels of methylation are observed when the cells are transfected with mGCM2 (Fig. 6c), in agreement with the strong increase in *mNr5a1* expression levels observed in MEF cells overexpressing mGCM2 (Fig. 5e). These data show that the mGCM proteins are not specifically involved in DNA demethylation and fit with the emerging view that gene expression is linked to DNA demethylation at the promoter and to DNA hypermethylation in the gene body^51^ (Fig. 6d). Our data are also in line with the recent hypothesis that transcription factors can bind demethylated as well as methylated DNA^52^. Finally, the high levels of expression of hNR5A1/mNR5A1 observed in endometriotic tissues are also linked to high levels of CpG methylation around exon 2 and low levels around the TSS^53^,^54^,^55^,^56^.

Overall, this data suggest that the *mGcm* genes induce the transcription of *mNr5a1* and this is associated with important changes in the DNA methylation profile at the *mNr5a1* locus.

## Discussion

In this study, we discover a molecular cascade required in the *Drosophila* female reproductive system that may be conserved in mammals. The *Drosophila* transcription factor Gcm is expressed during the development of the SC of the spermatheca and mutations or knock-down of Gcm inhibit the development of these cells, leading to female sterility. Gcm acts by targeting the ortholog of the *hNR5A1/mNR5A1* hormone receptor *Hr39*. Finally, the orthologous genes *mGcm1* and *mGcm2* are expressed in the mouse uterus, induce the expression of hNR5A1/mNR5A1 in human and murine cell lines, respectively and modify the DNA methylation profile of *mNr5a1*. This suggest that defects in the hGCM pathway may be associated with pathologies affecting women reproductive system.

### Common and tissue-specific features of the Gcm pathways

Gcm is required in the nervous, in the immune and in the reproductive systems. These Gcm dependent pathways display a common feature as, in all cases, a multipotent precursor gives rise to cells with different identities. In the nervous system, the neuroblast can produce glia or neurons, in the immune system the prohemocyte can produce plasmatocytes or crystal cells and in the spermatheca the MP can produce SC or LEC. Gcm is absolutely required to induce one fate over the other as *gcm* mutant animals lack glia and display supernumerary neurons^24^,^30^,^31^ and the number of plasmatocyte decreases whereas that of the crystal cells increases^36^,^38^. In the spermatheca, the absence of SC in homozygous *gcmGal4* animals is accompanied by an increase in LEC number (Fig. 2h, Supplemental Figure S4), suggesting that Gcm induces the differentiation of the SC at the expense of the LEC.

A second common feature between the three developmental events is the transient and early expression of Gcm. In the spermatheca, Gcm is expressed during the differentiation of the SC but no longer present in the adult SC. Similarly, Gcm is expressed early in the glial and in the hemocyte lineages but its transcripts are not detected in the mature cells^34^,^36^,^57^,^58^. Thus, the Gcm fate determinant provides a trigger that needs to be erased to allow terminal differentiation. In the nervous system, Gcm activates the transcription of its target gene *repo*, which remains expressed in glial cells until adulthood^59^. The Repo homeobox containing protein constitutes the pan-glial specific transcription factor that induces the expression of late glial genes, maintains the glial fate and actually contributes to Gcm degradation^57^,^59^ (Trebuchet, unpublished results). In the spermatheca, Gcm induces the expression of the Hr39 transcription factor that is required for SC formation and that remains expressed in those cells until adulthood^13^,^14^,^15^, Hr39 may hence play a maintenance role similar to that played by Repo in the glial cells. Recent data suggest that early and transient expression of fate determinants may be a general rule that allows stable and terminal cell differentiation. Interestingly, the *Drosophila* proneural transcription factor Atonal (Ato) is expressed early during photoreceptor differentiation but needs to be switched off for normal eye development^60^.

A third common feature between the three systems is the participation of the Notch pathway. In the spermatheca, the production of the SC from the initial MP encompasses three cells divisions. The first and third divisions involve the Notch pathway and trigger the differentiation of the LEP and the SC respectively^14^,^15^. In these two divisions, Notch is activate only in the cells that do not express Gcm suggesting that Notch and Gcm may interact negatively. Such negative interaction was previously reported during the differentiation of the adult sensory organ precursors (SOP). Constitutive activation of the Notch pathway in the SOP represses *gcm* expression and prevents the production of glial cells; accordingly, lack of Notch induces gcm expression and the production of glia at the expense of neurons^61^,^62^. Finally, during the development of the embryonic hemocytes, there is no report of interaction between Gcm and the Notch pathway, however Gcm is involved in plasmatocyte development and Notch in crystal cell development^34^,^36^,^63^. Importantly, several members of the Notch pathway are directly regulated by Gcm including the two ligands Serrate and Delta^39^, which suggests a strong interaction between Gcm and Notch that remains to be investigated.

Our work also highlights the cell-specific nature of the Gcm differentiation pathways: while the Gcm transcription factor is required to induce several cell identities, its downstream factors are cell-specific. Repo expression is absent in the spermatheca and Hr39 expression is absent in glial cells. Moreover, the overexpression of Gcm in the spermatheca does not activate Repo expression in those cells nor does Gcm overexpression in the embryonic nervous system activate Hr39 expression in that territory (data not shown). Thus, although ‘master regulators’ are considered as simple molecular switches, this represents an oversimplified view of cell differentiation. The activity of such potent transcription factors rather relies on the history of a given cell, that is, its specific transcriptional and epigenetic asset. For example, the ectopic expression of the famous *eyeless* master gene induces the formation of ectopic eyes on wings, legs and antennae^64^, while in the embryonic nervous system its ectopic expression alters the axonal wiring of the ventral nerve cord^65^.

Finally, the expression profile of Gcm gives an important insight on spermatheca differentiation. Our study shows that Gcm and Lz are co-expressed in the MP and that Gcm remains expressed exclusively in the SUP following the asymmetrical division of the MP whereas Lz is repressed in the SUP^14^. A comparable interaction between Gcm and Lz was observed during the differentiation of the embryonic hemocytes. Gcm is required for the differentiation of the plasmatocytes and Lz for the differentiation of the crystal cells^38^. Initially, Gcm is expressed in all prohemocytes but subsequently its expression fades away in the precursors of the crystal cells, which allows for the expression of Lz^34^,^37^,^38^. Thus, Gcm induces the plasmatocyte fate and inhibits the crystal cell fate through inhibition of Lz: as mentioned above, *gcm* mutant animals display supernumerary crystal cells and in addition ectopic Gcm expression in the crystal cell precursors using the *lzGal4* driver prevents the expression of Lz and converts cells into plasmatocytes^36^,^38^. We propose that in the spermatheca, Gcm is expressed at low levels in the MP where it cohabits with Lz, then its expression progressively rises in the SUP until its levels become sufficient to repress Lz expression in this cell. SUP cells that express low levels of Gcm adopt the LEC fate. The absence of Gcm binding sites at the *lz* locus and the known role of Gcm as an activator of transcription prompt us to speculate that Gcm represses Lz expression indirectly. The transcriptional repressor Tramtrack (Ttk)^66^,^67^ was already described as an inhibitor of Lz expression in the larval eye disc^68^, is a downstream target of Gcm^39^,^69^,^70^,^71^ and is expressed in the spermatheca^72^. Future studies will determine whether Ttk could act as the intermediary protein between Gcm and Lz inhibition.

### A conserved role for the Gcm family of proteins in the regulation of Hr39/Nr5a1 and fertility

Hr39 and NR5A1 transcription factors were proposed to share similar functions and to target similar genes for the development of specific secretory glands of the reproductive system (steroidogenic glands in mammals and spermathecae in *Drosophila*)^13^,^14^,^73^. Our study suggests that the control of Hr39 and NR5A1 by the GCM protein family is also conserved. Gcm controls female fertility due to its effects on the SC in the spermathecae, and the lack of SC is explained by the lack of induction of Hr39. This regulation is conserved in mammals with the *mGcm1* and *mGcm2* genes inducing mNR5A1 expression in MEF cells and being expressed in the reproductive system. This represents the first evidence of functional conservation for GCM proteins in similar biological systems of *Drosophila* and mammals.

Endometriosis^20^,^21^,^22^,^23^,^54^,^74^ is an oestrogen-dependent disorder defined by the ectopic growth of endometrium-like tissue (reviewed in ref. 75), which represents the leading cause of women infertility^76^,^77^,^78^. A major feature of endometriotic tissues is the overexpression of hNR5A1 and the modification of the *hNR5A1* DNA methylation profile in that tissue: the *hNR5A1* TSS is demethylated and the CpG island covering exon 2 is hypermethylated^22^,^53^,^54^,^55^; the present study shows that *mNr5a1* expression and its DNA methylation profile are regulated by the two mGCM proteins (Fig. 6d). This suggests that the GCM protein family could be involved in the pathogenesis of endometriosis. Over the past ten years, several studies aimed at identifying the molecular basis of endometriosis by comparing the transcriptomes of healthy endometrium to endometriotic tissue^56^,^79^,^80^,^81^,^82^,^83^,^84^. hGCM1 and hGCM2 did not come out in any of these studies. The large majority of these reports used micro-array to profile gene expression and both *hGCM1* and *hGCM2* were below the detection range in all studies even in healthy tissues whereas we detected *mGcm1* expression by qPCR and *mGcm2* expression by qPCR and *in situ* hybridisation. Several factors may explain the difficulty to identify the *hGCM* genes in those analyses, among them the known instability of their RNA and their potential transient expression (reviewed in ref. 85). This indicates that the study of GCM1 and GCM2 in endometriosis should be carried out using highly sensitive methods and possibly during the development of the disease to catch the transient presence of their transcripts.

Overall, our study suggests that the GCM regulatory network is robustly conserved and that *Drosophila* represents a model of choice to decipher this pathway in the reproductive system. Finally, this study indicates that the Gcm transcription factor has a much broader role than initially thought. We foresee that the deep analysis of its regulatory network will allow us to understand pleiotropic differentiation pathways and hence the role and mode of action of potent fate determinants.

## Materials and Methods

### Fly strain

Flies were raised on standard medium at 25 °C. The genotype and provenance of the strains are detailed in Supplemental experimental procedures.

### Fertility and egg laying assays

Fertility and egg laying assays are detailed in Supplemental experimental procedures. For fertility assays, the progeny produced in 12 days by 3 virgins of the indicated genotypes crossed with one male WT were counted and reported to number of progeny/female. For the egg laying assays, the number of eggs laid in 48 hrs by five females of the indicated genotypes crossed with ten males WT were counted and reported to number of eggs/females/days. The p-values were estimated after variance analysis using bilateral student test with equal variance.

### Immunolabelling

The spermathecae were labelled using standard immunolabelling protocol as described in ref. 39. The list of antibodies and the labelling protocol are detailed in in Supplemental experimental procedures.

### Secretory cell and basal cell counts

For each spermatheca, the Hnt/DAPI positive cells (SC) were counted from the stack of six focal plans taken at 3 μm interval in the middle of the spermatheca (the plan giving the largest cross-section of the spermatheca). This was repeated in at least six independent spermathecae for each genotype. The average number of SC and the s.e.m. are represented in Figs 2h and 4h. The p-values were estimated as described for the fertility assays.

### qPCR and luciferase assay in S2 cells

The transfection of S2 cells, the quantitative PCR (qPCR) and the luciferase assay were performed as described in Cattenoz *et al*.^39^ and detailed in Supplemental experimental procedures. Each experiment was carried out in triplicates.

### *In situ* hybridisation and RNA extraction from mouse uterus

RNA *in situ* hybridisation with digoxigenin-labelled probes for *mGcm2* transcripts was performed as described in Vernet *et al*.^86^ with slight modifications detailed in Supplemental experimental procedures. The qPCR were carried out on *C57BL/6* mouse uterus RNA extracted from 3 different animals with TRI reagent.

### Transfection and qPCR in mammalian cells

HeLa cells transfection was performed as described in Cattenoz *et al*.^39^ and MEF cells transfection was performed as detailed in Supplemental experimental procedures. 48 hrs after transfection, the cells were sorted according to GFP expression before RNA extraction. Reverse transcription and qPCR were carried out as described for the S2 cells with the primer pairs listed in Supplemental experimental procedures.

### Bisulfite sequencing in MEF cells

MEF cells transfected and sorted as described above were used to analyse the methylation profile of *mNr5a1* locus. The procedure is detailed in Supplemental experimental procedures. The loci of interest were then amplified by PCR, cloned and sequenced. At least 10 clones were sequenced per condition. The p-values were estimated after variance analysis using bilateral student test for paired samples.

## Additional Information

**How to cite this article**: Cattenoz, P. B. *et al*. An evolutionary conserved interaction between the Gcm transcription factor and the SF1 nuclear receptor in the female reproductive system. *Sci. Rep.* **6**, 37792; doi: 10.1038/srep37792 (2016).

**Publisher's note:** Springer Nature remains neutral with regard to jurisdictional claims in published maps and institutional affiliations.

## Supplementary Material

Supplementary Information
