# Supplementary material for: An evolutionary conserved interaction between the Gcm transcription factor and the SF1 nuclear receptor in the female reproductive system
Source: Sci Rep. 2016 Nov 25;6:37792. doi: 10.1038/srep37792 (PMC5122895; doi:10.1038/srep37792)
Supplement: Supplementary Information [file srep37792-s1.pdf]

Supplementary Information for:

An evolutionary conserved interaction between the Gcm transcription factor and the SF1 nuclear receptor in the female reproductive system

Pierre B. Cattenoz, Claude Delaporte, Wael Bazzi and Angela Giangrande*

Institut de Génétique et de Biologie Moléculaire et Cellulaire, IGBMC/CNRS/INSERM/UDS, BP 10142, 67404 ILLKIRCH, CU de Strasbourg, France.

***Corresponding author :**

Angela Giangrande,
Institut de Génétique et de Biologie Moléculaire et Cellulaire,
CNRS/INSERM/UDS, BP 10142,
67404 Illkirch Cedex,
CU de Strasbourg,
France.
angela@igbmc.fr

List of files:

Supplemental Figure S1: Expression levels of Gcm in gcm KD and gcm GOF spermatheca

Supplemental Figure S2: Expression and role of Gcm in the spermatheca development

Supplemental Figure S3: Expression and role of Gcm in the adult spermatheca

Supplemental Figure S4: lumen epithelial cells in gcm hypomorph spermatheca

Supplemental Experimental procedures

Supplemental Table S1: list of genes directly targeted by Gcm and expressed in spermatheca

Supplemental Figure S1, related to Figure 1

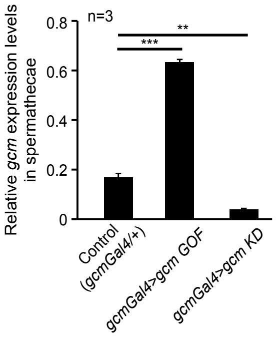

Supplemental Figure S1: Expression levels of Gcm in *gcm KD* and *gcm GOF* spermatheca
Expression levels of gcm in adult spermathecae measured by qPCR in *gcmGal4/+* (Control), *gcmGal4/+;UAS-gcm/+* (*gcm>gcm GOF*) and *gcmGal4/+;UAS-gcmRNAi/+* (*gcm>gcm KD*) animals. Each measurement was carried out in triplicate, normalised to the housekeeping genes *Gapdh* and *Act5C* and represented as described for **Figure 1a**. Each sample was prepared using at least 15 spermathecae.

Supplemental Figure S2, related to Figure 2

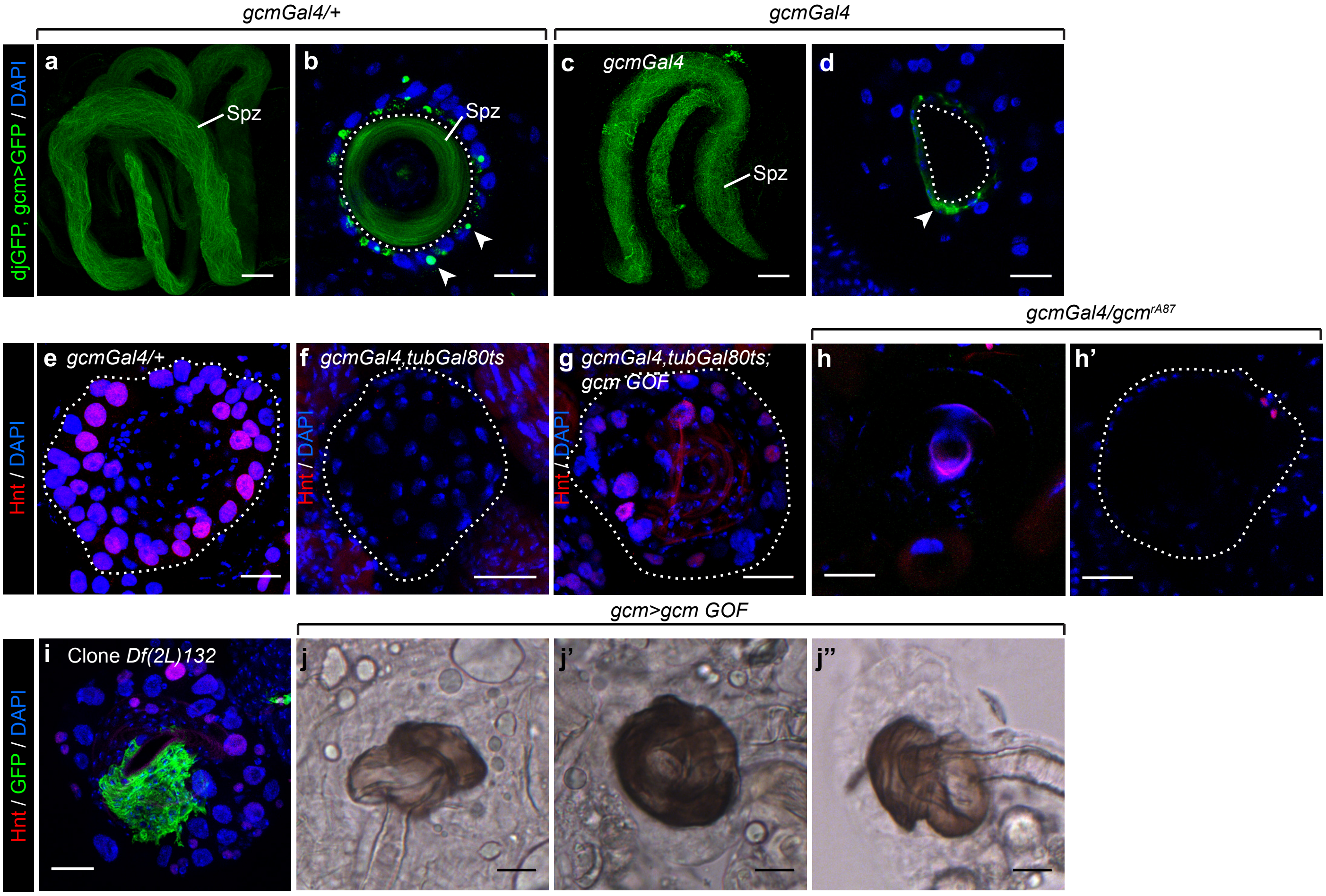

Supplemental Figure S2: Expression and role of Gcm in the spermatheca

a, c) Full confocal projections of *gcmGal4>GFP/+* **(a)** and *gcmGal4>GFP* homozygous **(c)** adult female seminal receptacles, carrying GFP positive spermatozoids (Spz) from *donjuanGFP* males (*djGFP*), labelled with anti-GFP (in green). The presence of the GFP signal in the seminal receptacles indicates that the females were inseminated. **b, d)** show single sections of the spermathecae that are attached to the seminal receptacles **(a, c)** labelled with anti-GFP (green) and DAPI (blue). The lumen of the spermatheca is outlined by a dashed line. Note the presence of GFP labelled spermatozoids in the *gcmGal4>GFP/+* spermatheca **(b)** and the absence of spermatozoids in the *gcmGal4>GFP* homozygous spermatheca **(d)**. The GFP positive cells (white arrowheads in **b and d**) indicate cells in which *gcm* promoter is activated (see **Supplemental Figure S3**). **e-g)** Full confocal projections of 1-day-old *gcmGal4/+* **(e)**, *gcmGal4/gcmGal4,tubGal80ts* (*gcmGal4,tubGal80ts*)**(f)** and *gcmGal4/gcmGal4,tubGal80ts;UAS-gcm* (*gcmGal4,tubGal80ts;gcm GOF*) **(g)** adult spermathecae from animals put at 29°C for 24 hrs after puparium formation to induce Gcm expression and labelled with anti-Hnt (Hnt, in red) and DAPI (blue). The dashed line outline the spermatheca. There are no SC in **(f)** whereas there are several SC (in red) in **(g)**. **h, h')** Single confocal sections of *gcmGal4/gcmrA87* adult spermathecae labelled with anti-Hnt (Hnt, in red) and DAPI (blue). The spermatheca is outlined with a dashed line in **(h')**. **i)** MARCM clone analysis of a null *gcm* mutation (*Df(2L)132*). The image represents the full projection of an adult spermatheca analysed by confocal microscopy. The clones are labelled with anti-GFP (green), anti-Hnt labelling is in red and DAPI in blue. **j-j'')** Images of spermathecae analysed by bright-field microscopy. The spermathecae were dissected from *gcmGal4/+;UAS-gcm/+* (*gcm>gcm GOF*) adult females (1 to 3-day-old).

Supplemental Figure S3, related to Figure 3

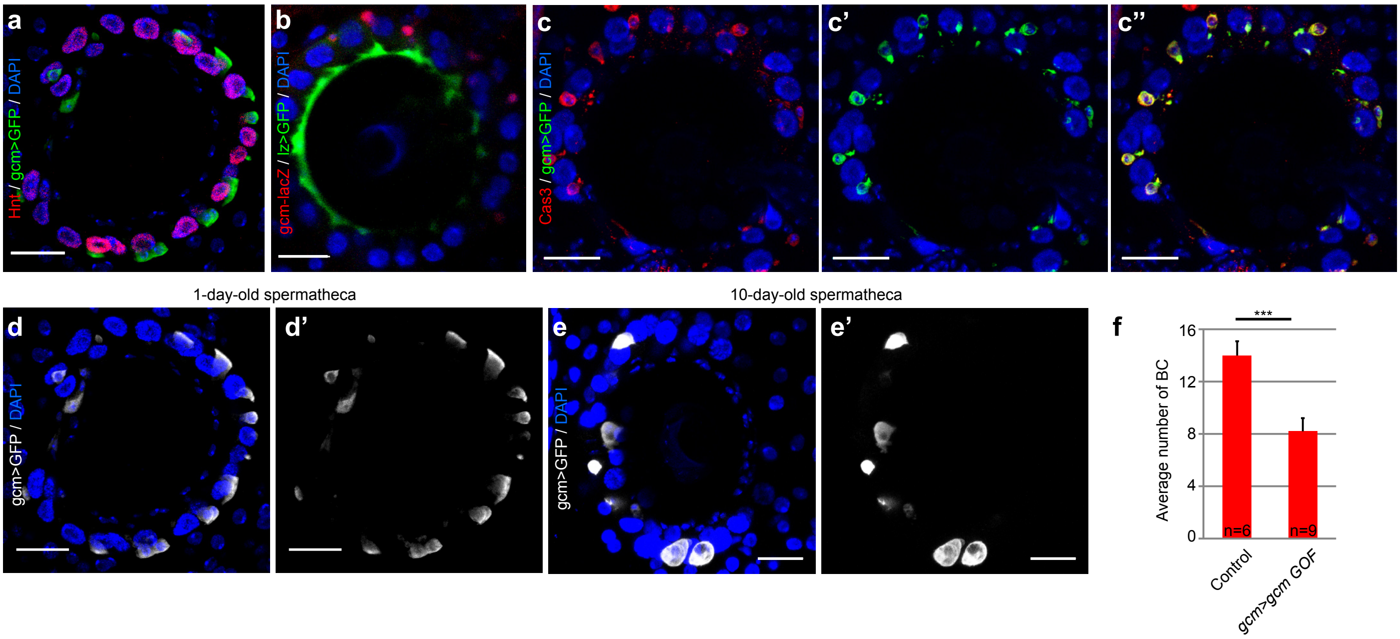

Supplemental Figure S3: Expression and role of Gcm in the spermatheca

a-c'') Single sections of adult spermathecae taken by confocal microscopy from adult control females (1 to 3-day-old). A gcmGal4,UAS-mCD8GFP/+ spermatheca **(a)** was labelled with DAPI (blue), anti-Hnt (Hnt, in red, secretory cell labelling) and anti-GFP (gcm>GFP, in green). A lzGal4,UAS-mCD8GFP/+;gcmrA87/+ adult spermatheca **(b)** was labelled with DAPI (blue), anti-βgal (gcm-lacZ, in red) and anti-GFP (lz>GFP, in green). A gcmGal4,UAS-mCD8GFP/+ adult spermatheca **(c-c'')** was labelled with anti-Caspase 3 (Cas3, in red), anti-GFP (gcm>GFP, in green) and DAPI (blue). **(c)** represents the overlay between anti-Cas3 and DAPI labelling, **(c')** between anti-GFP and DAPI labelling and **(c'')** between anti-Cas3, anti-GFP and DAPI labelling. **d-e')** Confocal projection of 1-day-old **(d, d')** and 10-day-old **(e, e')** gcmGal4,UAS-mCD8GFP/+ (gcm>GFP) adult spermathecae labelled with anti-GFP (gcm>GFP in grey) and DAPI (blue). **(d)** and **(e)** represent the overlay of DAPI and anti-GFP, **(d')** and **(e')** represent anti-GFP alone. **f)** Average number of basal cells (BC) counted in cross-sections of adult spermathecae of the indicated genotypes: gcmGal4/+ (Control) and gcmGal4/+;UAS-gcm/+ (gcm>gcm GOF). At least 6 spermathecae were analysed per genotype, the error bars and p-values are as described for **Figure 1a**.

Supplemental Figure S4, related to Figure 2

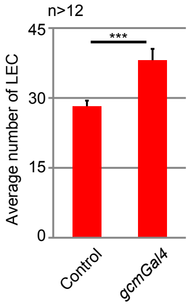

Supplemental Figure S4: lumen epithelial cells in gcm hypomorph spermatheca
Average number of lumen epithelial cells (LEC) counted in cross-sections of adult spermathecae of the indicated genotypes: *gcmGal4/+* (Control) and *gcmGal4* homozygous. At least 13 spermathecae were analysed per genotype, the error bars and p-values are as described for **Figure 1a**.

Supplemental experimental procedures

Fly strain

Flies were raised on standard medium at 25˚C. The following strains were used: the WT strain was *Oregon-R* (Bloomington #109612), *gcmGal4,UAS-mCD8GFP/CyO* [1] and *gcm*^{rA87}*/CyO* (Bloomington #5445) [2,3] were crossed with *sna*^{Sco}*/CyO,Tb*^{1} (Bloomington # 36335) to generate *gcmGal4,UAS-mCD8GFP/CyO,Tb*^{1} and *gcm*^{rA87}*/CyO,Tb*^{1} to identify homozygous and transheterozygous animals; the *gcm KD* was *P(TRiP.JF01075)attP2* (Bloomington #31519) and the *gcm GOF* was *UAS-gcmF18A* [4]; the efficiency of *gcm KD* and *gcm GOF* were verified by measuring the levels of Gcm expression in spermatheca using the driver *gcmGal4* **(Figure S1)**. Other strains used are *P[UAS-RedStinger]6* (*UAS-RFP* in the text, Bloomington #8547), *lzGal4,UAS-mCD8GFP* (Bloomington #6314), *UAS-Hr39* (FlyORF F00605) [5], *UAS-FLP:ubiFRT stop stinger III* (*g-trace* in the text) (Bloomington #28282). For the presence of spermatozoids in *gcmGal4/+* and *gcmGal4* homozygous spermathecae, 10 virgins of each genotype were mated for 3 days before dissection with 20 males *donjuanGFP* (*djGFP*, B# 5417) that express GFP in the spermatozoids [6] **(Figures S2a-d)**. For the rescue of the hypomorphic condition *gcmGal4* homozygous **(Figures S2e-g)**, *gcmGal4,UAS-mCD8GFP/CyO,Tb*^{1} animals were crossed with *Oregon-R*, *gcmGal4,tubGal80*^{ts}*/CyO,Tb*^{1} or *gcmGal4,tubGal80*^{ts}*/CyO,Tb*^{1}*;UAS-gcmF18A* [1]. Animals *gcmGal4,UAS-mCD8GFP/+*, *gcmGal4,tubGal80*^{ts}*/gcmGal4,UAS-mCD8GFP* and *gcmGal4,tubGal80*^{ts}*/gcmGal4,UAS-mCD8GFP;UAS-gcmF18A* were collected and incubated at 29°C for 24hrs APF and then put at 25°C until adulthood. For the MARCM clones, the strain *Df(2L)132/CyO* [7] was recombined with *FRT40A* (Bloomington #8212) to generate *FRT40A,Df(2L)132/CyO*; the strain *gcm*^{34}, produced by imprecise excision obtained upon mutagenesis [2,8], was recombined with *FRT40A* (Bloomington #8212) to generate *FRT40A,gcm*^{34}*/CyO*. The clones were generated as follow: the three strains *FRT40A*, *FRT40A,Df(2L)132/CyO* and *FRT40A,gcm*^{34}*/CyO* were crossed with *hsFLP,UAS-mCD8GFP;tubGal80,FRT40A;tubGal4* (Bloomington #42725), the progeny was then heat shocked at larval stage L3 at 37˚C for 3 hrs and the spermathecae were dissected in 1 to 3-day-old adults.

Fertility and egg laying assays

For fertility assays, three 1-day-old virgins of a given genotype were crossed with one 1-day-old male *Oregon-R* on standard medium at 25˚C. The cross was flipped every three days for twelve days. The progeny issued from the four bottles is counted at the adult stage. Each cross was replicated at least ten times and paired with a control (*Oregon-R* females). The average number of progeny per female of the ten replicates and standard error of the mean are represented in **Figure 1a**. For the egg laying assays, five 1-day-old females of the indicated genotypes were crossed with ten 1-day-old males *Oregon-R* for 3 days, then the flies were transferred to a cage to count the number of eggs laid over 48 hrs. The number of eggs was then reported to the number of females and the number of days in **Figure 2i**. The p-values were estimated after variance analysis using bilateral student test with equal variance (ns for not significant; "*" for p-value < 0.05, 0.01 <; "**" for p-value < 0.01, 0.001 <; "***" for p-value < 0.001)).

Immunolabelling

The spermathecae were dissected from 1 to 3-day-old females in PBS, fixed 20 min in 4% paraformaldehyde/PBS at room temperature (RT), rinsed 15 min in PTX (PBS, 0.3% triton-x100), incubated with blocking reagent (Roche) for 1 hr at RT, incubated overnight at 4˚C with primary antibodies diluted in blocking reagent, washed three times 10 min with PTX, incubated 1 hr at RT with the secondary antibodies, rinsed three times 10 min with PTX, incubated 30 min with DAPI (Sigma) diluted to 10^{-3} g/L in blocking reagent and mounted on slide in vectashield (Vector Laboratories). For immunolabelling of the pupal spermatheca, white pupae (0 hr after puparium formation (APF)) were collected and fixed at 24 hrs, 48 hrs or 72 hrs APF overnight in 4% paraformaldehyde at 4˚C. Then, the spermathecae were dissected and treated as the adult spermathecae. The slides were analysed by confocal microscopy (Leica, SP5) and the images treated with Fiji [9]. The following antibodies were used: rabbit anti-RFP 1/500 (abcam #ab62341), chicken anti-GFP 1/1000 (abcam #ab13970), mouse anti-Hnt 1/100 (DSHB 1G9), rabbit anti-βgal 1/500 (Cappel # 55976) and rabbit anti-Caspase 3 1/100 (abcam #13847). Secondary antibodies were: donkey anti-chicken coupled with FITC 1/400 (Jackson #703-095-155), donkey anti-rabbit coupled with Cy3 1/600 (Jackson #711-165-152), goat anti-mouse coupled with Alexa Fluor 647 1/400 (Jackson #115-605-166).

Secretory cell and basal cell counts

The spermatheca were dissected and labelled with anti-Hnt antibody and DAPI as described above. For each spermatheca, the Hnt/DAPI positive cells (secretory cells) were counted from the stack of six focal plans taken at 3µm interval in the middle of the spermatheca (the plan giving the largest cross-section of the spermatheca). This was repeated in at least six independent spermathecae for each genotype. The average number of secretory cells and the standard error of the mean are represented in **Figure 2h** and **Figure 4h**. The p-values were estimated after

variance analysis using bilateral student test with equal variance (ns for not significant; "*" for p-value <0.05, 0.01<, "**" for p-value <0.01, 0.001<, "***" for p-value < 0.001).

qPCR and luciferase assay in S2 cells

The transfection of S2 cells, the quantitative PCR (qPCR) and the luciferase assay were performed as described in Cattenoz et al. [10]. For the qPCR, 6 million S2 cells were plated per well in 6-well plates in 1.5 mL of Schneider medium complemented with 10% Fetal Calf Serum (FCS) and 0.5% penicillin and 0.5% streptomycin (PS). Cells were transfected 12 hrs after plating using the Effectene transfection reagent (Qiagen) using 2 µg of *pPac-gal4* vector and 1 µg of *pUAS-GFP* for the negative control (*ppacEmpty*) and 2 µg of *pPac-gcm* [11] and 1 µg of *4.3kb repo-GFP* (*repoGFP*) [12] for the *gcm* GOF assays (*ppacGcm*). After 48 hrs of transfection, the cells were sorted on a BD FACSAria according to GFP expression to obtain more than 80% of transfected cells in the sample. The RNA was then extracted using TRI reagent (Sigma), 1 µg of RNA per sample was DNAse treated with RNAse free DNAse 1 (Thermo Fisher) and reverse transcribed with Superscript II (Invitrogen). Quantitative PCR (qPCR) assays were performed on a lightcycler LC480 (Roche) with SYBR master (Roche) on the equivalent of 5 ng of reverse transcribed RNA with the primer pairs targeting *Hr39*, *hnt*, *Gapdh1* and *Act5c* listed below. Each PCR was carried out in triplicates on at least three biological replicates. The quantity of each transcript was normalized to the quantity of *Gapdh1* and *Act5c*. The p-values were measured comparing the control with the transfected cells using student test, the bars represent the standard error of the mean.

For the luciferase assay, WT and mutant reporters were built for each GBS at *Hr39* and *hnt* loci. Sense and anti-sense oligonucleotides covering the GBS in each gene were synthesized using flanking restriction sites for KpnI at the 5' extremity and NheI at the 3' extremity. Each pair of oligonucleotides was designed with the WT GBS and with a mutated GBS that is not bound by Gcm (mutated for nucleotides 2, 3, 6 and/or 7: list below, the restriction sites are indicated in capital letters). For each WT and mutant GBS, 2 µg of annealed oligonucleotide were digested with 20 U of KpnI (NEB # R3142S) and 20 U of NheI (NEB # R3131S) in Cutsmart buffer (NEB # B7204S) for 1 h 30 min at 37ºC. The digested double stranded probes were then cleaned and ligated in *pGL4.23* (Promega #E841A) (ratio plasmid:probe = 1:6). Transfections of *Drosophila* S2 cells were carried out in 12-well plates using Effectene transfection reagent (Qiagen #301427) according to manufacturer's instructions. Cells were transfected with 0.5 µg *pPac-lacZ*, 0.5 µg *pGL4.23* carrying the indicated GBS, 0.5 µg *pPac-gcm* [11] or 0.5 µg *pPac* [13]. 48 hrs after transfection, cells were collected, washed once in cold PBS and resuspended in 100 µL of lysis buffer (25 mM Tris-phosphate pH7.8, 2 mM EDTA, 1 mM DTT, 10% glycerol, 1% Triton X-100). The suspensions were frozen / thawed four times in liquid nitrogen and centrifuged 30 min at 4ºC at 13000 g. The Luciferase and βgal activities were measured in triplicates for each sample. For βgal measurements, 20 µL of lysate were mixed with 50 µL of β-galactosidase assay buffer (60 mM Na2PO4, 40 mM NaH2PO4, 10 mM KCl, 1 mM MgCl2, 50 mM β-mercaptoethanol) and 20 µL ONPG (4mg / mL) and incubated at 37ºC for 20 min. The reaction was stopped by adding 50 µL 1M Na2CO3 and the DO at 415 nm was measured. For Luciferase activity, 10 µL of protein lysate were analysed on an opaque 96-well plate (Packard instrument # 6005290) with a Berthold Microluminat LB96P Luminometer by injecting 50 µL of luciferase buffer (20 mM Tris-phosphate pH 7.8, 1 mM MgCl2, 2.5 mM MgSO4, 0.1 mM EDTA, 0.5 mM ATP, 0.5 mM luciferine, 0.3 mM coenzyme A, 30 mM DTT). For both βgal and Luciferase assays, background levels were estimated using lysate from not transfected S2 cells. The relative Luciferase activities were calculated as follow: first the background was subtracted from each value, then the average values of the technical triplicate were calculated. From there, the Luciferase activity of each sample was normalized to the βgal activity (Luciferase activity / βgal activity) to correct for transfection efficiency variability and the ratio (Luciferase with Gcm / Luciferase without Gcm) was calculated. For each WT and mutant GBS, biological triplicates were carried out.

In situ hybridisation and RNA extraction from mouse uterus

RNA *in situ* hybridisation with digoxigenin-labelled probes for *mGcm2* transcripts was performed as described in Vernet et *al.* [14] with slight modifications. Cryosections (10 µm sections) of mouse (*C57BL/6*) uterus were labelled with sense or anti-sense probes targeting *mGcm2* (only the anti-sense probe is shown). The probes were synthesized from the clone 40054293 inserted into *pCR-BluntII-TOPO* using the Ribo-probe *in vitro* transcription system (Promega).

To assess *mGcm1*, *mGcm2* and *mNr5a1* levels of expression in uterus (**Figure 5f**), the RNA was extracted from the uterus of *C57BL/6* using TRI reagent (Sigma) and the qPCR were carried out as described below for mammalian cells. The levels were estimated from 3 different animals.

Transfection and qPCR in mammalian cells

HeLa cells were plated in 6-well plates, 400,000 cells per well, in 1.6 mL of DMEM medium complemented with 5% FCS and gentamycin. Cells were transfected 12 hrs after plating using Effectene transfection reagent (Qiagen). Briefly, 1 µg of *pCIG* vector, 1 µg of *pCIG* vector expressing mGCM1 (*pCIG-mGcm1)* [15] or 1 µg of *pCIG* vector

expressing mGCM2 (*pCIG-mGcm2)* were mixed with 100 µL of EC buffer and 8 µL of enhancer, incubated 5 min at room temperature, then 10 µL of Effectene were added and the mix was incubated at room temperature for 20 min. 200 µL of DMEM medium + 5% FCS + gentamycin were added to the mix before spreading it on the cells. 48 hrs after transfection, the RNA was extracted using TRI reagent (Sigma).

MEF cells were plated in 6-well plates, 400,000 cells per well, in 1.6 mL of DMEM medium (4.5g/L glucose) complemented with 10% FCS, 1% sodium pyruvate and 0.5% penicillin and 0.5% streptomycin. Cells were transfected 12 hrs after plating using Effectene transfection reagent (Qiagen). Briefly, 1 µg of *pCIG* vector, 1 µg of *pCIG* vector expressing mGCM1 (*pCIG-mGcm1)* [15] or 1 µg of *pCIG* vector expressing mGCM2 (*pCIG-mGcm2)* were mixed with 100 µL of EC buffer and 8 µL of enhancer, incubated 5 min at room temperature, then 10 µL of Effectene were added and the mix was incubated at room temperature for 20 min. 200 µL of DMEM medium + 5% FCS + gentamycin were added to the mix before spreading it on the cells. After 48 hrs of transfection, the cells were sorted on a BD FACSAria according to GFP expression (the *pCIG* vectors express GFP constitutively) to obtain more than 80% of transfected cells in the sample. The RNA was then extracted using TRI reagent (Sigma).

Reverse transcription and qPCR were carried out as described for the S2 cells with the primer pairs listed below. The quantity of each transcript was normalized to the quantity of the housekeeping genes *Glyceraldehyde 3 phosphate dehydrogenase* (*Gapdh*) and *Actin Beta (ActnB)*.

Bisulfite sequencing in MEF cells

MEF cells transfected and sorted as described above were used to analyse the methylation profile of *mNr5a1* locus. After sorting, the cells were incubated 1.5 hrs at 37°C in 20 mM EDTA, 10 mM Tris pH 8.0, 200 mM NaCl, 0.2% Triton X-100 and 100 mg/mL proteinase K and centrifuged at room temperature for 5 min at 14000 rpm. The DNA was precipitated from the supernatant by adding 1 vol. of isopropanol and 1/20 vol. of 4M NaCl, incubating the sample overnight at -20°C and centrifugation at 4°C for 25 min at 14000 rpm. The DNA pellet was suspended in demineralized water and treated with RNAse A for 1 hr at 37°C. Then 500 ng of DNA was digested with BamH1 restriction enzyme and converted with bisulfite using EZ DNA methylation Direct Kit (ZYMO #D5020) according to the manufacturer instruction. The loci of interest were then amplified by PCR using the ZymoTaq DNA polymerase (ZYMO #E2001) and the primers indicated below. The PCR products were cloned in pGEM-T Easy vector and sequenced by Sanger sequencing (GATC Biotech). At least 10 clones were sequenced per condition. The p-values were estimated after variance analysis using bilateral student test for paired samples (ns for not significant; "*" for p-value <0.05, 0.01<, "**" for p-value <0.01, 0.001<, "***" for p-value < 0.001).

List of primers:

specie	gene	Forward	Reverse
drosophila	Act5c	GCCAGCAGTCGTCTAATCCA	GACCATCACACCCTGGTGAC
drosophila	Gapdh1	CCCAATGTCTCCGTTGTGGA	TGGGTGTCGCTGAAGAAGTC
drosophila	Hr39	CCCAACTGGCTTTTGGGTAAC	AGAGGTGTCGTTGATGCAGTT
drosophila	hnt	TTTCAACGGGAACCAAGCCT	AGCATTTTTCCAACGGCTAGTT
drosophila	lz	CACCTATGTCACCATCCGGG	ACCTTGATGGCTTTGGCGTA
human	ACTNB	ATGATGATATCGCCGCGCTC	TCGATGGGGTACTTCAGGGT
human	GAPDH	GAGAAGGCTGGGGCTCATTT	AGTGATGGCATGGACTGTGG
human	NR5A1	AGCTGCAAGGGCTTCTTCAA	GCTTGTACATCGGCCCAAAC
human	NR5A2	GAGTCCAGGGAAAGACTTGCT	GCCTTGGGAAGGACACATCA
drosophila	Hr39GB S1mut	gagaGGTACCatattcttgtaattaaaaagttagtcgttgcttatgcatg cttatcttccGCTAGCgaga	tctcGCTAGCggaagataagcatgcataagcaacgactaactttttaa ttacaagaatatGGTACCtctc
drosophila	Hr39GB S1wt	gagaGGTACCatattcttgtaattaaaaagttatgcgggacttatgcat gcttatcttccGCTAGCgaga	tctcGCTAGCggaagataagcatgcataagtcccgcataactttttaa ttacaagaatatGGTACCtctc
drosophila	Hr39GB S2mut	gagaGGTACCagtgggcttaggatcttcgcacaacgactctccggc gggcatatcacgtcGCTAGCgaga	tctcGCTAGCgacgtgatatgcccgccggagagtcgttgtgcgaag atcctaagcccactGGTACCtctc
drosophila	Hr39GB S2wt	gagaGGTACCagtgggcttaggatcttcgcaccccgcatctccggc gggcatatcacgtcGCTAGCgaga	tctcGCTAGCgacgtgatatgcccgccggagatgcggggtgcgaa gatcctaagcccactGGTACCtctc
drosophila	hntGBS mut	gagaGGTACCctggcttttaatgtatatttacaacgacttgccattacca tcattatattGCTAGCgaga	tctcGCTAGCaatataatgatggtaatggcaagtcgttgtaaatataca ttaaaagccagGGTACCtctc
drosophila	hntGBS wt	gagaGGTACCctggcttttaatgtatatttaacacgcattgccattacca tcattatattGCTAGCgaga	tctcGCTAGCaatataatgatggtaatggcaatgcgtgttaaatataca ttaaaagccagGGTACCtctc
mouse	ActnB	TACCAACTGGGACGACATGGAGAA	GCTCGAAGTCTAGAGCAACATAGC
mouse	Gapdh	TGAACGGGAAGCTCACTGG	TCCACCACCCTGTTGCTGTA
mouse	Gcm1	AAAGCCAGACAGAAGCAGCA	GCTCGCCTTTGGACTGGAAA

mouse	Gcm2	CACAGCGGATACCCTGTCAC	CAGCCGTGCTATTGAGGTGT
mouse	Nr5a1	CCGAGAGTCAGAGCTGCAAA	CATTCGATCAGCACGCACAG
mouse	Nr5a2	CAGTTCGATCAGCGGGAGTT	TGGGTAGTTGCAAACCGTGT
mouse	Msx1	CCGAAAGCCCCGAGAAACTA	CGCTCGGCAATAGACAGGTA
mouse bisulfite	Nr5a1 CpG exon 2	GTTTTGTTTTAGAGGAAGGGAATGA	CCCCCAAAACAATCCAACTATATAC
mouse bisulfite	Nr5a1 CpG TSS	GGTATTTTTAAATTGGATTAGTAAA	ATACAAAAAATAAAAAACAAACTAC

Supplemental bibliography

1 Soustelle, L. & Giangrande, A. Novel gcm-dependent lineages in the postembryonic nervous system of Drosophila melanogaster. *Developmental dynamics : an official publication of the American Association of Anatomists* **236**, 2101-2108, doi:10.1002/dvdy.21232 (2007).

2 Vincent, S., Vonesch, J. L. & Giangrande, A. Glide directs glial fate commitment and cell fate switch between neurones and glia. *Development* **122**, 131-139 (1996).

3 Jones, B. W., Fetter, R. D., Tear, G. & Goodman, C. S. glial cells missing: a genetic switch that controls glial versus neuronal fate. *Cell* **82**, 1013-1023 (1995).

4 Bernardoni, R., Miller, A. A. & Giangrande, A. Glial differentiation does not require a neural ground state. *Development* **125**, 3189-3200 (1998).

5 Bischof, J. *et al.* A versatile platform for creating a comprehensive UAS-ORFeome library in Drosophila. *Development* **140**, 2434-2442, doi:10.1242/dev.088757 (2013).

6 Santel, A., Winhauer, T., Blumer, N. & Renkawitz-Pohl, R. The Drosophila don juan (dj) gene encodes a novel sperm specific protein component characterized by an unusual domain of a repetitive amino acid motif. *Mech Dev* **64**, 19-30 (1997).

7 Lane, M. E. & Kalderon, D. Genetic investigation of cAMP-dependent protein kinase function in Drosophila development. *Genes Dev* **7**, 1229-1243 (1993).

8 Bernardoni, R., Vivancos, V. & Giangrande, A. glide/gcm is expressed and required in the scavenger cell lineage. *Developmental biology* **191**, 118-130 (1997).

9 Schindelin, J. *et al.* Fiji: an open-source platform for biological-image analysis. *Nat Methods* **9**, 676-682, doi:10.1038/nmeth.2019 (2012).

10 Cattenoz, P. B. *et al.* Functional Conservation of the Glide/Gcm Regulatory Network Controlling Glia, Hemocyte, and Tendon Cell Differentiation in Drosophila. *Genetics* **202**, 191-219, doi:10.1534/genetics.115.182154 (2016).

11 Miller, A. A., Bernardoni, R. & Giangrande, A. Positive autoregulation of the glial promoting factor glide/gcm. *The EMBO journal* **17**, 6316-6326, doi:10.1093/emboj/17.21.6316 (1998).

12 Laneve, P. *et al.* The Gcm/Glide molecular and cellular pathway: new actors and new lineages. *Dev Biol* **375**, 65-78, doi:10.1016/j.ydbio.2012.12.014 (2013).

13 Krasnow, M. A., Saffman, E. E., Kornfeld, K. & Hogness, D. S. Transcriptional activation and repression by Ultrabithorax proteins in cultured Drosophila cells. *Cell* **57**, 1031-1043 (1989).

14 Vernet, N. *et al.* Retinoic acid metabolism and signaling pathways in the adult and developing mouse testis. *Endocrinology* **147**, 96-110, doi:10.1210/en.2005-0953 (2006).

15 Soustelle, L. *et al.* Neurogenic role of Gcm transcription factors is conserved in chicken spinal cord. *Development* **134**, 625-634, doi:10.1242/dev.02750 (2007).

Table S1: list of genes directly targeted by Gcm and expressed in spermatheca

Genes directly targeted by Gcm (DamID screen, Cattenoz et al., 2016)	FBgn ID	Expression level in wild type spermatheca (average of two replicates, Allen and Spradling, 2008)
abd-A	FBgn0000014	1829,095
Abd-B	FBgn0000015	677,4245
ago	FBgn0041171	322,1235
AGO1	FBgn0262739	285,8825
Alh	FBgn0261238	131,733
Amun	FBgn0030328	238,319
aop	FBgn0000097	253,3475
apt	FBgn0015903	80,7979
Asph	FBgn0034075	154,041
Atet	FBgn0020762	395,57
Atg18a	FBgn0035850	1107,745
Atg5	FBgn0029943	148,614
Atg9	FBgn0034110	425,416
Atpalpha	FBgn0002921	1462,565
att-ORFB	FBgn0067782	88,1157
aux	FBgn0037218	594,9015
Axn	FBgn0026597	157,274
B52	FBgn0004587	1488,09
babos	FBgn0034724	135,052
bbg	FBgn0087007	2010,615
ben	FBgn0000173	2030,455
Best1	FBgn0040238	455,301
beta-Man	FBgn0037215	450,122
brat	FBgn0010300	99,5663
bt	FBgn0005666	697,293
bur	FBgn0000239	285,802
caps	FBgn0023095	253,692
CASK	FBgn0013759	316,754
cbt	FBgn0043364	1183,14
cbx	FBgn0011241	243,0905
CenG1A	FBgn0028509	469,813
CG10055	FBgn0037482	169,1075
CG10098	FBgn0037472	572,0075
CG10178	FBgn0032684	167,13
CG10195	FBgn0032787	93,28125
CG10311	FBgn0038420	2515,81
CG10465	FBgn0033017	1077,596
CG10939	FBgn0010620	1299,975
CG1103	FBgn0037235	218,371
CG1109	FBgn0046222	125,185
CG1124	FBgn0037290	1162,495
CG11279	FBgn0036342	393,922

CG11537	FBgn0035400	875,004
CG11576	FBgn0039882	353,8115
CG11920	FBgn0039274	167,485
CG11961	FBgn0034436	910,8705
CG12007	FBgn0037293	373,9425
CG12054	FBgn0039831	158,3635
CG12547	FBgn0250830	192,1735
CG12948	FBgn0037739	103,7605
CG12991	FBgn0030847	3409,88
CG13096	FBgn0032050	149,327
CG13366	FBgn0025633	156,689
CG13384	FBgn0032036	190,9485
CG13506	FBgn0034723	558,174
CG13728	FBgn0036716	108,20965
CG13907	FBgn0035173	398,4595
CG14040	FBgn0031676	348,458
CG14442	FBgn0029893	146,667
CG14478	FBgn0028953	278,568
CG14687	FBgn0037835	151,309
CG14764	FBgn0033236	191
CG14995	FBgn0035497	259,762
CG15523	FBgn0039727	220,99
CG1598	FBgn0033191	502,09
CG1677	FBgn0029941	365,829
CG17002	FBgn0033122	167,88
CG17266	FBgn0033089	87,80935
CG2145	FBgn0030251	1066,94
CG2162	FBgn0035388	258,393
CG2182	FBgn0037360	527,9685
CG2201	FBgn0032955	355,782
Cg25C	FBgn0000299	277,816
CG2617	FBgn0032877	136,6545
CG2811	FBgn0035082	307,318
CG30015	FBgn0050015	209,106
CG30069	FBgn0050069	413,4735
CG30080	FBgn0050080	126,7545
CG30159	FBgn0050159	541,558
CG30344	FBgn0050344	1228,855
CG3036	FBgn0031645	986,6955
CG30463	FBgn0050463	279,3715
CG30497	FBgn0050497	1359,405
CG31365	FBgn0051365	152,792
CG31368	FBgn0051368	103,878
CG31457	FBgn0051457	101,66915
CG31637	FBgn0051637	670,041
CG31650	FBgn0031673	186,0595
CG32264	FBgn0052264	555,1535
CG32344	FBgn0052344	155,0555

CG32486	FBgn0266918	493,772
CG32521	FBgn0052521	535,1485
CG32640	FBgn0052640	923,0625
CG33158	FBgn0053158	87,4974
CG3402	FBgn0035148	208,413
CG34317	FBgn0085346	87,7564
CG3558	FBgn0025681	252,7325
CG3702	FBgn0031590	652,7435
CG3760	FBgn0022343	781,8275
CG3781	FBgn0029853	214,034
CG3792	FBgn0031662	549,183
CG3857	FBgn0023520	127,3298
CG40006	FBgn0058006	139,7875
CG42238	FBgn0250867	93,85015
CG42389	FBgn0259735	342,392
CG43658	FBgn0263706	207,06
CG43675	FBgn0263750	1012,1355
CG4452	FBgn0035981	1170,395
CG45186	FBgn0266696	1132,88
CG4747	FBgn0043456	501,1685
CG5087	FBgn0035953	159,6335
CG5270	FBgn0037897	112,0487
CG5346	FBgn0038981	600,2225
CG5445	FBgn0030838	220,76
CG5789	FBgn0039207	121,699
CG5867	FBgn0027586	4016,45
CG6023	FBgn0030912	81,5793
CG6040	FBgn0038679	267,17
CG6145	FBgn0033853	1071,365
CG6276	FBgn0038316	302,3585
CG6398	FBgn0030870	568,0415
CG7009	FBgn0038861	102,41315
CG7029	FBgn0039026	257,271
CG7139	FBgn0027532	682,843
CG7337	FBgn0031374	583,441
CG7378	FBgn0030976	140,6595
CG7806	FBgn0032018	303,3565
CG7987	FBgn0038244	143,767
CG8188	FBgn0030863	81,53025
CG8507	FBgn0037756	467,826
CG9281	FBgn0030672	1409,69
CG9300	FBgn0036886	109,11155
CG9650	FBgn0029939	383,3235
CG9701	FBgn0036659	4153,89
CG9780	FBgn0037230	80,07805
CG9799	FBgn0038146	113,65085
CG9801	FBgn0037623	238,3685
cindr	FBgn0027598	146,3195

CkIalpha	FBgn0015024	922,2765
coro	FBgn0265935	1181,195
Cortactin	FBgn0025865	131,3395
corto	FBgn0010313	1077,61
COX7C	FBgn0040773	4380,115
cpo	FBgn0263995	1444,055
Crag	FBgn0025864	349,2645
crb	FBgn0259685	351,172
CrebA	FBgn0004396	1595,61
CREG	FBgn0025456	303,495
crq	FBgn0015924	404,3545
Cy	FBgn0283531	296,1175
D2hgdh	FBgn0023507	271,794
dally	FBgn0263930	584,2865
dap	FBgn0010316	271,126
DCTN1-p150	FBgn0001108	324,751
Den1	FBgn0033716	157,937
Diap1	FBgn0260635	2749,735
dj-1beta	FBgn0039802	431,59
Dl	FBgn0000463	171,823
DMAP1	FBgn0034537	115,33905
Dmtn	FBgn0037443	557,328
DnaJ-1	FBgn0263106	6758,065
dnr1	FBgn0260866	222,918
dnt	FBgn0024245	97,99755
DOR	FBgn0035542	486,865
dos	FBgn0016794	339,02
dpy	FBgn0053196	1570,855
drk	FBgn0004638	1060,39
drongo	FBgn0020304	672,84
Dscam1	FBgn0033159	84,1407
Dys	FBgn0260003	3099,89
E2f1	FBgn0011766	2029,85
Edem1	FBgn0023511	323,928
edl	FBgn0023214	291,1635
eIF-2gamma	FBgn0263740	942,966
eIF-3p40	FBgn0022023	1929,29
eIF-4a	FBgn0001942	6124,24
eIF5B	FBgn0026259	685,0165
Eip63E	FBgn0005640	1166,54
Eip74EF	FBgn0000567	447,344
Elal	FBgn0013949	157,7995
EloA	FBgn0039066	187,6915
Ent1	FBgn0031250	799,01
Epac	FBgn0085421	963,4245
Esp	FBgn0013953	314,4645
ex	FBgn0004583	299,0635
Fas2	FBgn0000635	526,6475

Fas3	FBgn0000636	852,5175
FER	FBgn0000723	131,925
fwd	FBgn0004373	609,5835
fz2	FBgn0016797	433,395
g	FBgn0001087	131,3705
Gale	FBgn0035147	1014,0025
GalT1	FBgn0053145	84,23735
Gcn5	FBgn0020388	250,3245
glec	FBgn0015229	511,565
Glut4EF	FBgn0267336	164,9285
Gmd	FBgn0031661	485,4145
Gug	FBgn0010825	512,232
gw	FBgn0051992	895,2145
h	FBgn0001168	5930,74
hang	FBgn0026575	152,127
hdly	FBgn0038842	3544,44
heph	FBgn0011224	213,0225
hh	FBgn0004644	477,587
Hmgcr	FBgn0263782	95,68645
hng2	FBgn0037634	94,9183
HnRNP-K	FBgn0267791	340,9705
hppy	FBgn0263395	298,003
Hr39	FBgn0261239	772,797
Hrb87F	FBgn0004237	1038,851
Hs6st	FBgn0038755	153,8465
Hsc70-4	FBgn0266599	12644,75
Hsc70Cb	FBgn0026418	641,0515
hth	FBgn0001235	726,5975
Idh	FBgn0001248	3326,935
if	FBgn0001250	275,283
ImpL2	FBgn0001257	131,3735
InR	FBgn0283499	144,56
Inx3	FBgn0265274	594,4525
jar	FBgn0011225	1007,4675
jbug	FBgn0028371	732,383
jigr1	FBgn0039350	157,6645
jumu	FBgn0015396	144,6845
Kap-alpha1	FBgn0024889	484,8155
KLHL18	FBgn0037978	107,88
ko	FBgn0020294	707,648
koi	FBgn0265003	595,6345
ksh	FBgn0040890	669,6045
l(1)10Bb	FBgn0001491	420,4975
l(2)k12914	FBgn0263852	3145,32
l(3)neo38	FBgn0265276	196,739
l(3)psg2	FBgn0035617	113,9855
Lac	FBgn0010238	1923,46
lama	FBgn0016031	1087,34

lbl	FBgn0008651	1484,115
loco	FBgn0020278	455,156
lola	FBgn0283521	828,4015
lsn	FBgn0260940	280,314
luna	FBgn0040765	94,43645
mbc	FBgn0015513	1122,725
mei-P26	FBgn0026206	91,289
Meltrin	FBgn0265140	103,1934
Mes2	FBgn0037207	275,719
Mes-4	FBgn0039559	84,78605
mew	FBgn0004456	866,7745
mfas	FBgn0260745	581,6975
mgl	FBgn0261260	716,8855
mib1	FBgn0263601	303,742
mino	FBgn0027579	219,214
Mlp84B	FBgn0014863	606,092
mrj	FBgn0034091	1724,175
mRpL53	FBgn0050481	196,9175
msi	FBgn0011666	148,8165
msps	FBgn0027948	463,6045
mtd	FBgn0013576	865,4615
Mvl	FBgn0011672	354,929
nahoda	FBgn0034797	106,39835
Ndae1	FBgn0259111	103,5275
Ndfip	FBgn0052177	1977,31
ND-MNLL	FBgn0029971	1807,635
neur	FBgn0002932	96,80635
NKAIN	FBgn0085442	152,775
nkd	FBgn0002945	104,84615
noc	FBgn0005771	422,5045
osp	FBgn0003016	405,4145
oys	FBgn0033476	922,7415
p130CAS	FBgn0035101	165,78
par-1	FBgn0260934	93,9153
par-6	FBgn0026192	488,499
Pdk1	FBgn0020386	1056,35
peb	FBgn0003053	842,197
Pep	FBgn0004401	1102,5555
Pfrx	FBgn0027621	180,896
PGAP3	FBgn0033088	217,1185
Pgd	FBgn0004654	541,2645
PH4alphaEFB	FBgn0039776	8051,64
pho	FBgn0002521	549,4505
ph-p	FBgn0004861	171,9425
Pino	FBgn0016926	1174,95
pio	FBgn0020521	496,5205
Pka-C1	FBgn0000273	84,73255
Pli	FBgn0025574	137,3795

pnr	FBgn0003117	1827,98
pnt	FBgn0003118	2030,275
pnut	FBgn0013726	579,796
Pp2C1	FBgn0022768	194,47
Ppa	FBgn0020257	585,5515
prtp	FBgn0030329	530,5815
psq	FBgn0263102	152,468
Ptp61F	FBgn0267487	1716,745
Ptp99A	FBgn0004369	251,6575
Pu	FBgn0003162	933,49
Pura	FBgn0035802	1020,345
px	FBgn0003175	273,0125
pyd	FBgn0262614	1562,895
Pym	FBgn0034918	149,3055
qsm	FBgn0028622	359,591
r	FBgn0003189	116,765
Rab5	FBgn0014010	1099,214
Rab7	FBgn0015795	3783,915
Rac2	FBgn0014011	607,2435
RanBP3	FBgn0039110	406,878
RapGAP1	FBgn0264895	448,128
Ras85D	FBgn0003205	545,923
Rbcn-3B	FBgn0023510	367,62
Rcd4	FBgn0032034	188,79
rdx	FBgn0264493	516,5365
retn	FBgn0004795	3303,91
rho	FBgn0004635	442,029
rho-7	FBgn0033672	257,817
RhoGEF3	FBgn0264707	141,212
rin	FBgn0015778	215,582
RnrS	FBgn0011704	222,299
robl	FBgn0024196	1104,58
Roc2	FBgn0044020	706,575
RpL17	FBgn0029897	8210,895
Rpn9	FBgn0028691	497,4765
S	FBgn0003310	166,3285
S6k	FBgn0283472	926,1285
scaf	FBgn0033033	689,6155
schlank	FBgn0040918	459,5625
scny	FBgn0260936	356,0485
Sdc	FBgn0010415	700,8805
Sema-5c	FBgn0250876	104,0513
Sh3beta	FBgn0035772	2314,405
shot	FBgn0013733	2537,105
Shroom	FBgn0085408	774,104
sip3	FBgn0039875	491,6555
Sirup	FBgn0031971	101,1519
siz	FBgn0026179	817,2835

Slip1	FBgn0024728	406,9095
slv	FBgn0025469	462,7635
smid	FBgn0016983	178,432
smo	FBgn0003444	197,764
Snx3	FBgn0038065	1012,6165
Socs36E	FBgn0041184	586,758
Socs44A	FBgn0033266	195,805
SoxN	FBgn0029123	254,3775
Spn	FBgn0010905	145,3065
SPoCk	FBgn0052451	138,0055
Spps	FBgn0039169	95,9651
spri	FBgn0085443	85,04325
sqd	FBgn0263396	607,8055
Srp19	FBgn0015298	952,216
Ssadh	FBgn0039349	257,847
Ssdp	FBgn0011481	422,85
ssp3	FBgn0032723	85,136
Stat92E	FBgn0016917	1965,775
sty	FBgn0014388	153,1655
Su(Tpl)	FBgn0014037	1132,55
Sur-8	FBgn0038504	123,2645
sws	FBgn0003656	428,5395
Taf4	FBgn0010280	609,292
Tango11	FBgn0050404	511,76
TBCB	FBgn0034451	295,5295
Ten-m	FBgn0004449	243,6385
TER94	FBgn0261014	1483,58
Tina-1	FBgn0035083	3143,01
Tis11	FBgn0011837	453,957
tmod	FBgn0082582	247,0415
tna	FBgn0026160	921,1385
toc	FBgn0015600	207,3655
TRAM	FBgn0040340	9577,845
trbl	FBgn0028978	287,3145
Trim9	FBgn0051721	132,807
trn	FBgn0010452	127,328
Tsp42Ea	FBgn0029508	2371,83
Tsp66E	FBgn0035936	528,7745
twin	FBgn0011725	371,3215
ush	FBgn0003963	536,773
Usp10	FBgn0052479	847,299
Usp47	FBgn0016756	809,1385
Usp8	FBgn0038862	137,4285
uzip	FBgn0004055	270,623
vih	FBgn0264848	235,558
vimar	FBgn0022960	159,4325
wake	FBgn0266418	251,123
wgn	FBgn0030941	111,2778

wun	FBgn0016078	293,285
Wwox	FBgn0031972	128,813
X11L	FBgn0026313	118,71785
yin	FBgn0265575	128,719
Zasp52	FBgn0265991	750,3135
ZIPIC	FBgn0039740	81,0034
Zir	FBgn0031216	265,127
Zn72D	FBgn0263603	227,223
Zpr1	FBgn0030096	220,2635
